# CQRM: A Composite Quantile Regression Model for Differential Expression Analysis of Non-Coding RNA Count Data

**DOI:** 10.3390/ncrna12050039

**Published:** 2026-10-13

**Authors:** Zhangchi Xu, Rong Pan, Tianzhou Ma

**Affiliations:** 1 Department of Epidemiology and Biostatistics, University of Maryland, College Park, MD 20742, USA; xzc0907@umd.edu (Z.X.); rpan0805@umd.edu (R.P.); 2 Department of Mathematics, University of Maryland, College Park, MD 20742, USA

**Keywords:** differential expression analysis, non-coding RNA, RNA-seq, scRNA-seq, quantile regression

## Abstract

**Background/Objectives:** Non-coding RNAs (ncRNAs) play important roles in various biological processes, primarily via regulating gene expression at multiple levels. Nevertheless, compared to protein-coding genes, ncRNAs are relatively understudied. They are characterized by low expression, large variation, and more dynamic expression across different tissues and cell types, which creates unique challenges in differential expression (DE) analysis. **Methods:** We propose the CQRM, an adaptive multi-quantile testing framework for ncRNA count data that incorporates jittering to accommodate discrete counts, combines dependent evidence across quantiles, and selects the most informative quantile subset for distinguishing conditions. **Results:** The CQRM captures diverse expression differences, including changes in variability, skewness, and modality beyond mean shifts. Model-based simulations showed that the CQRM improved the detection of differentially expressed ncRNAs while controlling the false discovery rate relative to existing DE tools, especially when DE genes exhibited multimodal expression patterns and counts were low. Critical ncRNAs and biologically interpretable targeted pathways were identified when we further applied our method to a Smart-seq-total single-cell RNA-seq dataset and a human organ developmental RNA-seq dataset. **Conclusions:** CQRM complements mean-based DE methods by capturing heterogeneous ncRNA expression changes, supporting the identification of candidate ncRNA biomarkers and regulatory processes relevant to development and disease.

## 1. Introduction

Non-coding RNAs (ncRNAs) comprise a diverse class of RNA molecules that lack protein-coding potential but play essential roles in gene regulation and cellular function. These include long non-coding RNAs (lncRNAs), microRNAs (miRNAs), small nuclear RNAs (snRNAs) and circular RNAs (circRNAs), among others. Increasing evidence demonstrates that ncRNAs are involved in chromatin organization, transcriptional regulation, RNA processing, translation, and intercellular signaling and contribute to numerous physiological and pathological processes across plants and animals [1,2,3,4,5]. Dysregulation of ncRNA expression has been implicated in a wide range of diseases, including cancer, neurodegenerative disorders, cardiovascular diseases, and psychiatric conditions, making ncRNAs attractive biomarkers and therapeutic targets [1,2,3,4,5].

Compared with coding genes, many ncRNAs exhibit lower expression levels, stronger tissue- and cell-type specificity, reduced evolutionary conservation, and greater expression heterogeneity [6,7]. These features suggest specialized regulatory functions but also create substantial analytical challenges for transcriptomic studies. The widespread adoption of bulk and single-cell RNA sequencing (scRNA-seq) technologies in recent decades has enabled transcriptome-wide profiling of ncRNA expression and stimulated growing interest in identifying differentially expressed (DE) ncRNAs that are associated with biological processes and disease phenotypes. However, DE analysis of ncRNAs is considerably more challenging than that of protein-coding genes as many ncRNA species are characterized by low abundance, high sparsity, substantial biological variability, and multimodal expression distributions [8,9]. These characteristics are often biologically meaningful and may reflect heterogeneous transcriptional regulation, cell-state specificity, or context-dependent expression patterns. Consequently, DE analysis may manifest not only shifts in mean expression levels but also changes in variability, skewness, sparsity, or other distributional properties. Representative examples of such ncRNA expression distributions are shown in Figure S1.

For count data from RNA-seq or scRNA-seq, the existing DE methods can be broadly classified into parametric count-based methods, nonparametric methods, and zero-inflation-adjusted methods (Table 1). Parametric methods such as edgeR [10] and DESeq2 [11] model gene expression counts using the negative binomial (NB) distribution and primarily focus on detecting differences in mean expression levels between conditions. Limma-voom [12] transforms count data and employs empirical Bayes moderated linear models to improve variance estimation and inference, particularly in small-sample settings. Although these methods have demonstrated excellent performance for mRNA analyses, the regime in which many ncRNAs are measured differs from the one they were designed for. Counts are low, zero fractions are high, and normalization factors are estimated largely from a small number of abundant transcripts; under those conditions the negative-binomial mean–variance relationship is estimated from little information, and the performance drops accordingly [9].

Nonparametric DE methods such as the Wilcoxon rank-sum test [13], SAMSeq [14], and NOISeq [15] relax distributional assumptions and often provide improved robustness in heterogeneous settings. The Wilcoxon test has been highlighted in recent comparative studies as a more robust approach for DE analysis with better false discovery rate (FDR) control and power performance [16]. However, rank-based methods generally focus on differences in central tendency and may have limited sensitivity when DE arises through more complex distributional changes in variability, skewness, sparsity, or multimodality. Benchmarking studies have accordingly reported reduced sensitivity and unstable FDR control for low-abundance transcripts, including lncRNAs and other ncRNAs, when standard pipelines and normalization are applied in this regime [9]. For scRNA-seq data, several zero-inflated methods have been proposed, including zero-inflated negative binomial (ZINB) models [17] and a zero-inflated quantile regression model ZIAQ [18]. While these methods improve modeling of sparse expression data, they primarily focus on dropout mechanisms. Dedicated DE methods that were specifically designed for ncRNAs remain relatively limited. Existing methods such as lncDIFF [19] target count-transformed continuous data and have demonstrated improved performance for lowly expressed lncRNAs but generally remain tied to specific distributional assumptions or particular ncRNA classes. Given the growing recognition of ncRNAs as critical regulators in health and disease, there remains a pressing need for flexible statistical frameworks that are capable of accommodating the diverse and heterogeneous expression patterns observed across ncRNA species.

In this paper, we propose a composite quantile regression model (CQRM) for DE analysis of ncRNA sequencing data. Unlike conventional DE methods that primarily target differences in mean expression, the CQRM integrates information across multiple quantiles of the expression distribution, enabling the detection of a wider range of DE patterns. By avoiding restrictive parametric assumptions, the CQRM accommodates the heterogeneous and often non-standard expression distributions that are commonly observed in ncRNAs. In addition, its regression-based framework naturally accommodates complex experimental designs and covariate adjustment.

**Table 1:** Summary of major DE methods used to model count data from RNA-seq or scRNA-seq.

Category	Method	Main Model	Main Characteristics	Reference
Parametric	DESeq2	Negative binomial (NB)	size-factor normalization; NB GLM, Wald test.	[11]
	edgeR	NB	robust NB GLM, likelihood ratio test.	[10]
	limma-voom	Transform + linear	voom transformation; linear model + eBayes shrinkage.	[12]
Nonparametric	Wilcoxon test	No distribution assumption	rank-based	[13]
	SAMSeq	No distribution assumption	rank-based and resampling-based test.	[14]
	NOISeq	No distribution assumption	nonparametric and data-adaptive test.	[15]
Zero-inflation (scRNA-seq)	ZINB-WaVE	Zero-inflated NB	both dimension reduction and signal extraction	[17]
	ZIAQ	Zero-inflated quantile regression	zero-inflation adjusted quantile regression model + Fisher’s *p*-value combination.	[18]

## 2. Results

### 2.1. Simulation

#### 2.1.1. Simulation Settings

We compared the CQRM with competing methods using a model-based simulation with prespecified differentially expressed genes (DEGs) and parameters estimated from real data. Specifically, we generated counts for G=2000 genes under two conditions, each with n=100 samples, using mixtures of negative binomial (NB) distributions implemented in the scDD package [20]. The NB mean and dispersion parameters were sampled from real brain lncRNA data [21], with mean counts in (0.5,10] for half of the genes (a low count level reflecting low-abundance ncRNAs), and TCGA KIRC protein-coding gene data [22], with mean counts in (10,50] for the remaining half (a medium count level). We designated 20% of the genes as true DEGs. These genes followed either a *bimodal* pattern, with a single mode in each condition and differences arising from mean shifts, or a *multimodal* pattern, with differences in both modality and mean expression [20]. This design allowed us to assess whether quantile-based methods provide greater advantages for multimodal expression patterns. The remaining 80% of genes were non-differentially expressed. Weak, moderate, and strong signals were defined by $|\log_{2}FC|∼U\left(0.25,0.75\right)$, U(0.75,1.25), and U(1.25,2.00), respectively. For sensitivity analysis, we varied the proportion of multimodal DEGs and the sample size. To assess the impact of severe sparsity, we conducted additional simulations using a zero-inflated negative binomial (ZINB) model, varying the probability of an excess zero across 0.2, 0.4, 0.6, 0.8, and 0.9, and compared the CQRM with competing methods.

We compared ten methods: DESeq2, edgeR, limma-voom, Wilcoxon, NOISeq, ZIAQ, CQRM, CQRM-Best, CQRM-Region and lncDIFF. We evaluated both detection power and false discovery rate (FDR) control. Detection power was assessed as the proportion of true DE genes among the top-ranked genes identified by each method. To evaluate error control, we computed the empirical FDR among genes declared significant at nominal FDR thresholds of 0.1, 0.05, 0.01, and 0.001. All the results were summarized over 50 independent simulation replicates.

#### 2.1.2. Simulation Results

**Figure 1:**
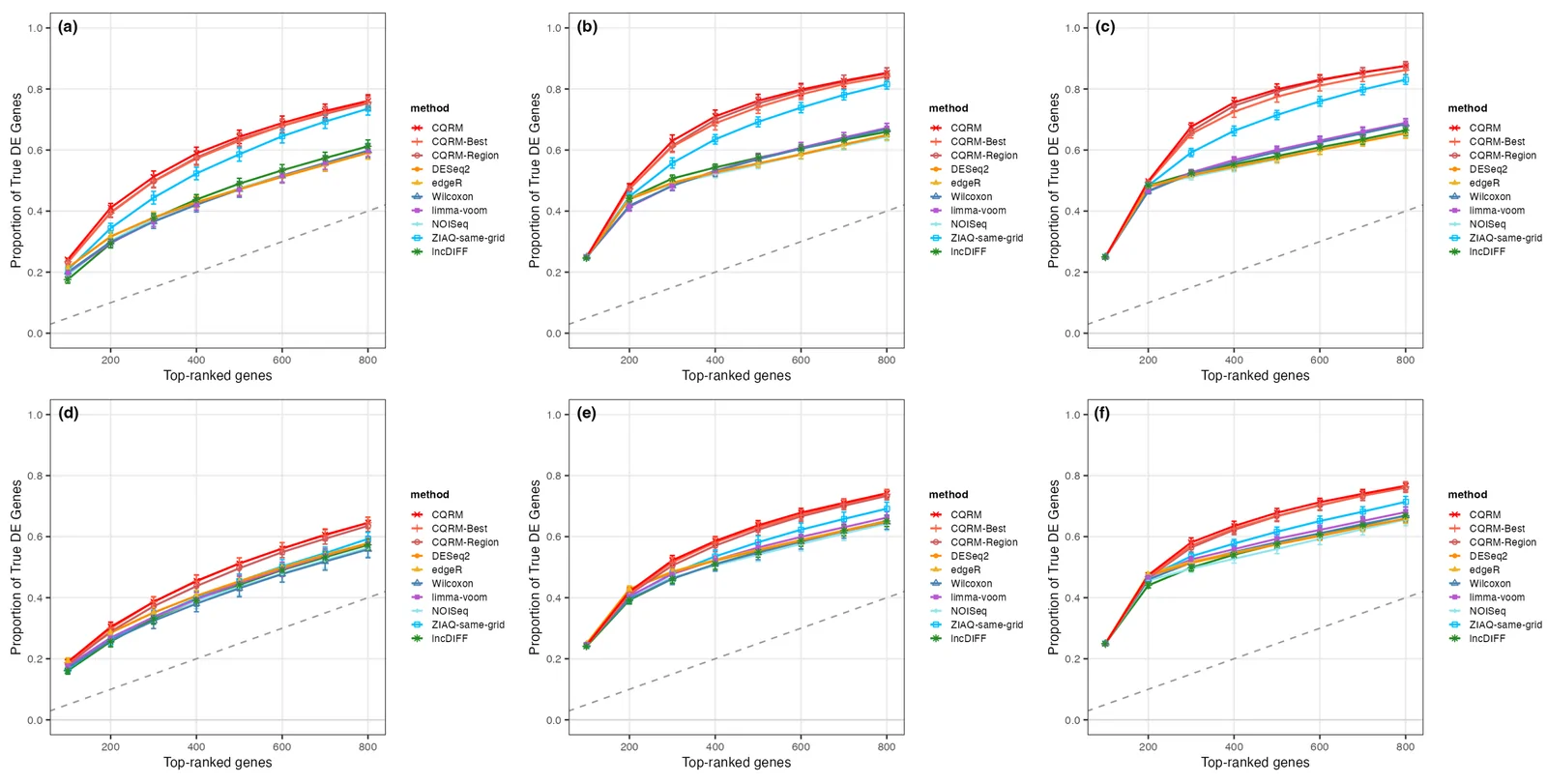
Model-based simulation: proportion of all true DEGs recovered among the top-ranked genes for ten methods. Bimodal DE = 50%, multimodal DE = 50%. (**a**–**c**): Moderate-count regime at the weakest, median and strongest signal levels. (**d**–**f**): Low-count regime, same three levels.

**Figure 2:**
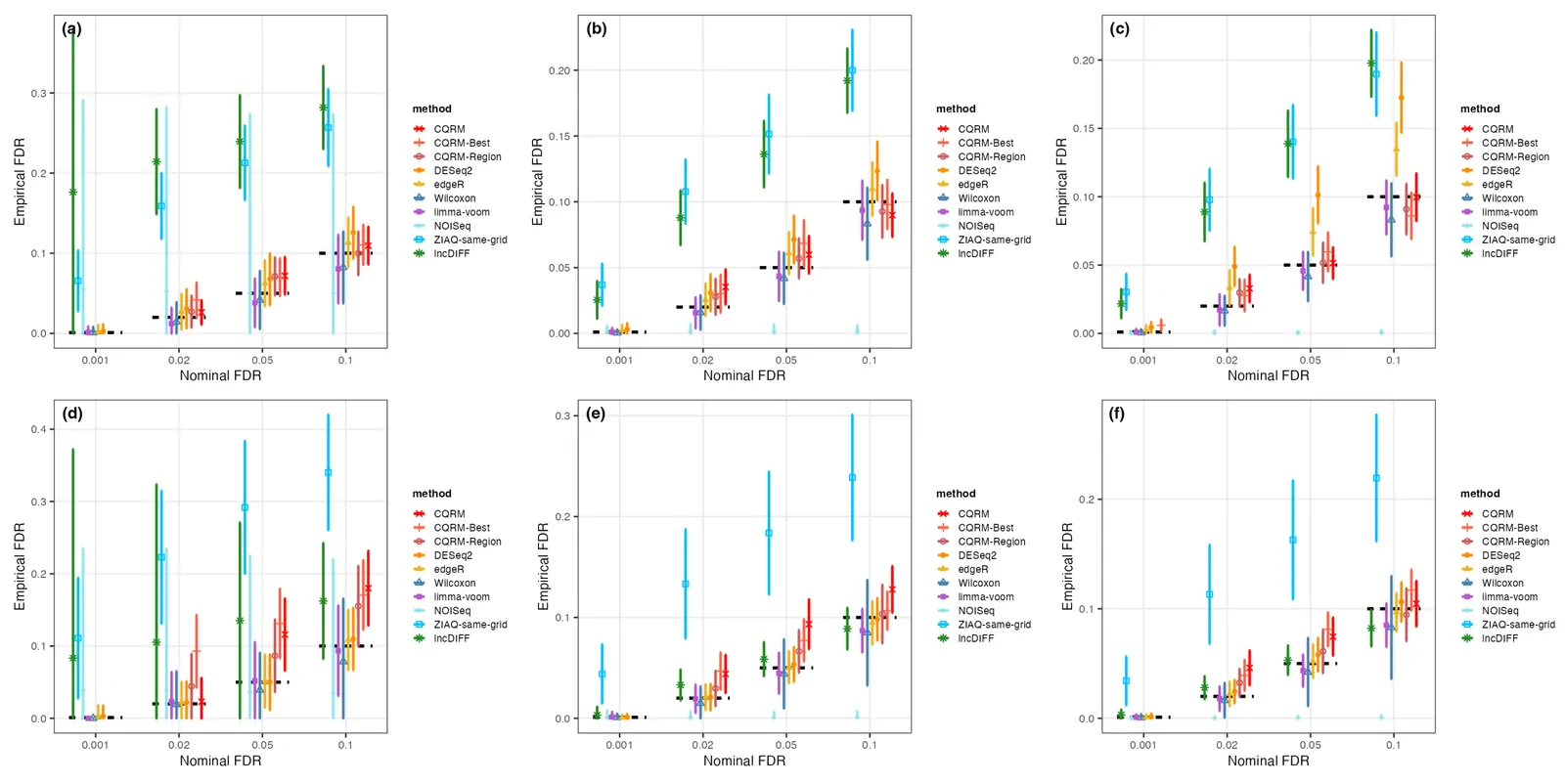
Model-based simulation: empirical FDR against nominal FDR for ten methods. Bimodal DE = 50%, multimodal DE = 50%. (**a**–**c**): Moderate-count regime at the weakest, median and strongest signal levels. (**d**–**f**): Low-count regime, same three levels.

The CQRM and its variants generally recovered more true DE genes among the top-ranked genes than competing methods (Figure 1) while showing no clear FDR inflation (Figure 2) for all six scenarios. For the low-count regime, the strength of the CQRM (e.g., over ZIAQ) became even more pronounced. ZIAQ had strong power but tended to inflate FDR, while other methods, including DESeq2, edgeR, Wilcoxon, limma-voom, NOISeq and lncDIFF, were generally underpowered. The advantage remained but was less pronounced as fewer (25%) DEGs followed multimodal patterns, reflecting the strength of the CQRM particularly for complex DEGs with multimodal distributions (Figures S2 and S3). When generating the counts from ZINB with varying probability of excess zeros, the CQRM performed well under relatively moderate zero inflation. As zero inflation became more severe (0.6–0.8), gene recovery declined across the methods, including the CQRM (Figure S4a,b). These results support the CQRM’s utility under moderate sparsity while confirming that jittering alone cannot overcome the information loss caused by extreme sparsity.

We also performed a sensitivity analysis of (1) quantile τ selection for the CQRM method (Figure S5) and (2) the impact of sample size on the CQRM and other methods (Figure S6). For quantile selection, we recommend avoiding extreme quantiles and overly narrow grids. Grids with intermediate coverage and moderate size, e.g., the grid used here τ∈{0.2,0.4,0.6,0.8} or tertiles τ∈{0.25,0.5,0.75}, achieved optimal power while maintaining strict empirical FDR control at or below the nominal 0.05 threshold. As sample size increased, the advantage of quantile regression-based methods, including the CQRM and its variants, over other methods became more pronounced. We recommended more than 50 samples per group to maintain the high power of the CQRM while keeping FDR under control.

**Computational cost.** The computational cost of the CQRM is linear in the number of genes since each gene is tested independently and grows superlinearly in the sample size because a quantile regression is refitted for each of the *m* jittered replicates at each τ. It is accordingly one to two orders of magnitude slower than mean-based methods, while its peak memory is comparable to or lower than theirs, so the cost is in time rather than memory. The other quantile regression-based methods, like ZIAQ, are slower at moderate and large sample sizes. For large-scale analysis (e.g., single-cell RNA-seq data), the asymptotic version CQRM-Approx can potentially replace the computationally heavy jittering process but at the risk of zero counts collapsing or becoming inflated. The runtime and memory for all the methods along the sample-size axis are summarized in Figure S7 and Tables S1 and S2.

### 2.2. Case Study 1: DE Analysis of Different ncRNA Species Across Human Cell Lines

To evaluate the performance of the CQRM on diverse non-coding RNA species, we analyzed the broad-spectrum RNA sequencing dataset GSE151334 generated using the scRNA-seq tool Smart-seq-total [23]. Unlike conventional RNA-seq protocols that primarily capture mRNAs, Smart-seq-total profiles the entire cellular RNA content, including mRNAs, lncRNAs, miRNAs, snoRNAs, snRNAs, and other non-coding RNA species. We analyzed the total RNAomes of human primary dermal fibroblasts, HEK293T cells (human embryonic kidney cell line), and MCF7 cells (human breast cancer cell line) from the original study. Cells with fewer than 2000 detected genes or more than two million sequencing reads were excluded. RNAs lacking annotation were removed prior to analysis. Overall, expression levels are low in this dataset, with median counts for both ncRNA and total RNA close to zero and mean counts below 50. Pairwise differential expression analyses were then performed among fibroblasts, HEK293T, and MCF7 cells using edgeR, Wilcoxon, and the CQRM.

**Figure 3:**
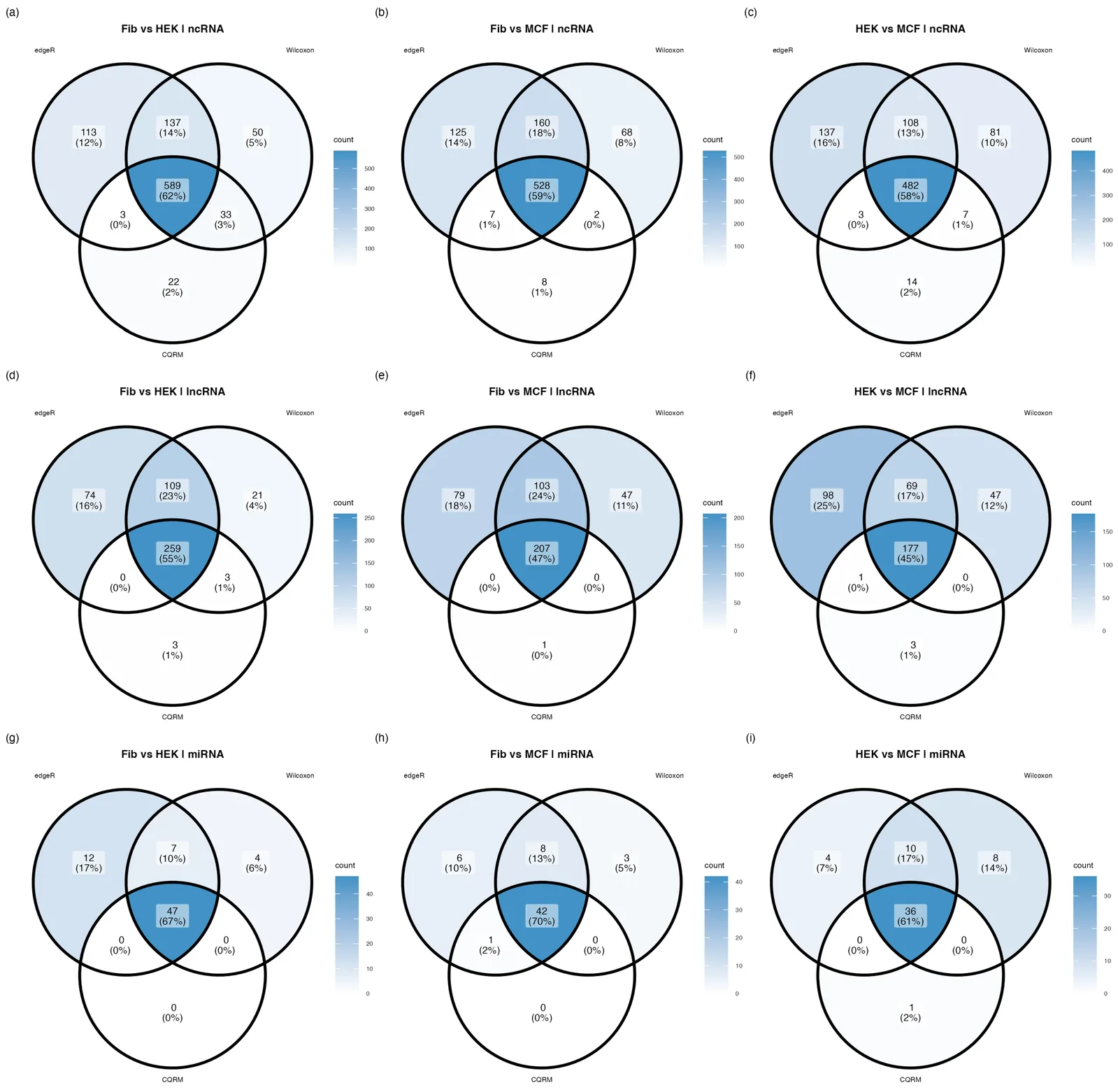
Overlap of ncRNAs called differentially expressed at Benjamini–Hochberg adjusted p<0.05 by CQRM, edgeR and Wilcoxon. Columns are the three pairwise cell-line comparisons; rows are all annotated ncRNAs (**a**–**c**), lncRNA (**d**–**f**) and miRNA (**g**–**i**); snoRNA and snRNA are in Figure S8.

Because the true DE status is unknown in real data, we take agreement among methods as the reference: genes identified by all three methods at Benjamini–Hochberg adjusted p<0.05 form a consensus core. Across the three pairwise comparisons, the CQRM identified 647, 545 and 506 significant ncRNAs for fibroblast vs. HEK, fibroblast vs. MCF, and HEK vs. MCF, respectively (Figure 3a–c). The CQRM showed a higher proportion of significant ncRNAs shared by all three methods. This pattern was consistent across lncRNAs and miRNAs (Figure 3d–i; snoRNA and snRNA are shown separately in Figure S8).

We annotated the top 100 ncRNAs identified by the CQRM for each cell line comparison using LncBook v2.0 [24], NCBI Gene, GeneCards [25] and the literature (Tables S3–S5). Several highly ranked ncRNAs were consistent with published cell-type-related biology. *DSCAM-AS1* and *LINC00052* ranked first and second in both MCF-7 comparisons but were absent from the fibroblast–HEK293T comparison. Both have reported roles in estrogen-responsive breast cancer biology [26,27]. *KCNQ1OT1* appeared among the top 100 in both fibroblast comparisons and has been implicated in fibroblast proliferation, migration, and extracellular-matrix accumulation [28]. *RN7SK*, ranked 17th in the fibroblast–HEK293T comparison, has been linked experimentally to apoptosis in human embryonic kidney cells [29]. These annotations demonstrate that the CQRM prioritizes biologically coherent cell-type-relevant signals while also identifying many poorly characterized ncRNAs for which the current annotation resources remain limited.

### 2.3. Case Study 2: DE Analysis of lncRNA During Human Organ Development

In the second example, we used the CQRM to analyze a large-scale developmental transcriptome dataset spanning multiple organs and developmental stages [21]. Raw bulk RNA-seq count data spanning developmental stages of multiple human organs (brain, cerebellum, heart, kidney, liver, ovary, and testis) were obtained from LncExpDB (https://ngdc.cncb.ac.cn/lncexpdb/downloads; accession E-MTAB-6814, accessed on 27 June 2025)). For the present analysis, only annotated lncRNAs were retained. LncRNAs with detectable expression in fewer than 20% of the samples were excluded to reduce the influence of extremely sparse transcripts. The developmental stages were dichotomized into early and late developmental periods. Samples labeled as weeks post-conception or days post-birth were classified as early-stage, whereas samples labeled as years post-birth were classified as late-stage. Organs with insufficient sample sizes or highly imbalanced numbers of early- and late-stage samples (heart, kidney, liver, and ovary) were excluded from the downstream analyses. Consequently, differential expression analyses were conducted for brain, cerebellum, and testis. DE analyses were carried out between early and late developmental stages for each organ using the edgeR, Wilcoxon, ZIAQ and CQRM methods.

**Figure 4:**
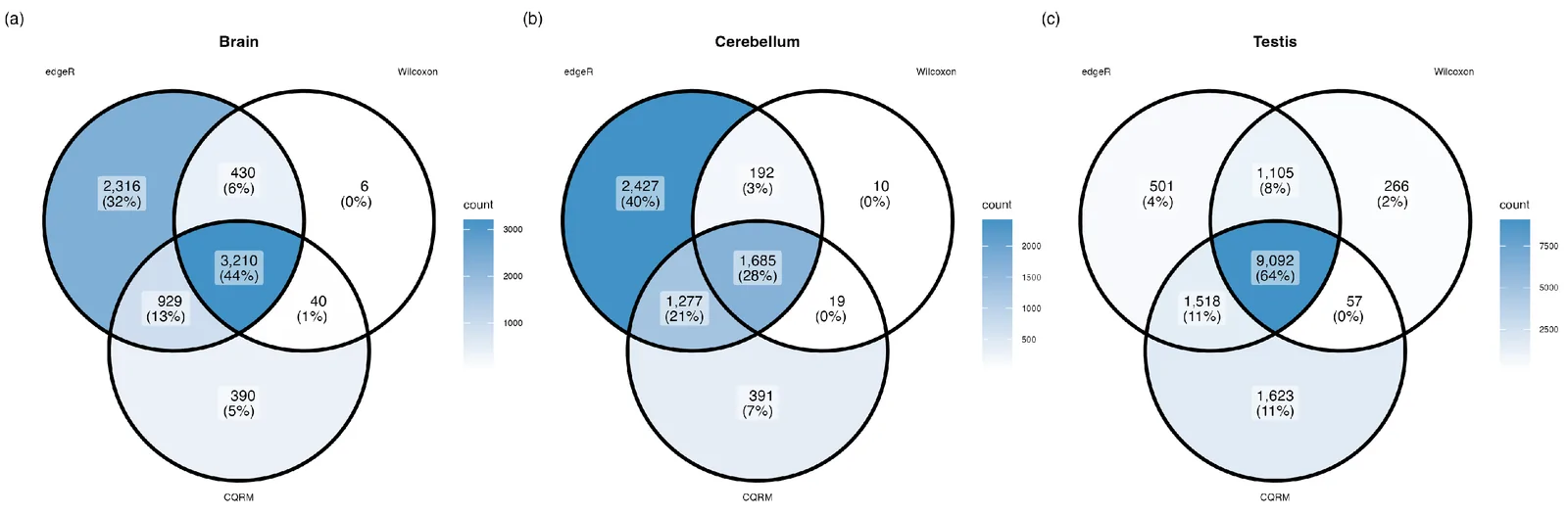
Case study 2: overlap of lncRNAs called differentially expressed at Benjamini–Hochberg adjusted p<0.05 by CQRM, edgeR and Wilcoxon between early and late developmental stages in brain (**a**), cerebellum (**b**) and testis (**c**).

**Figure 5:**
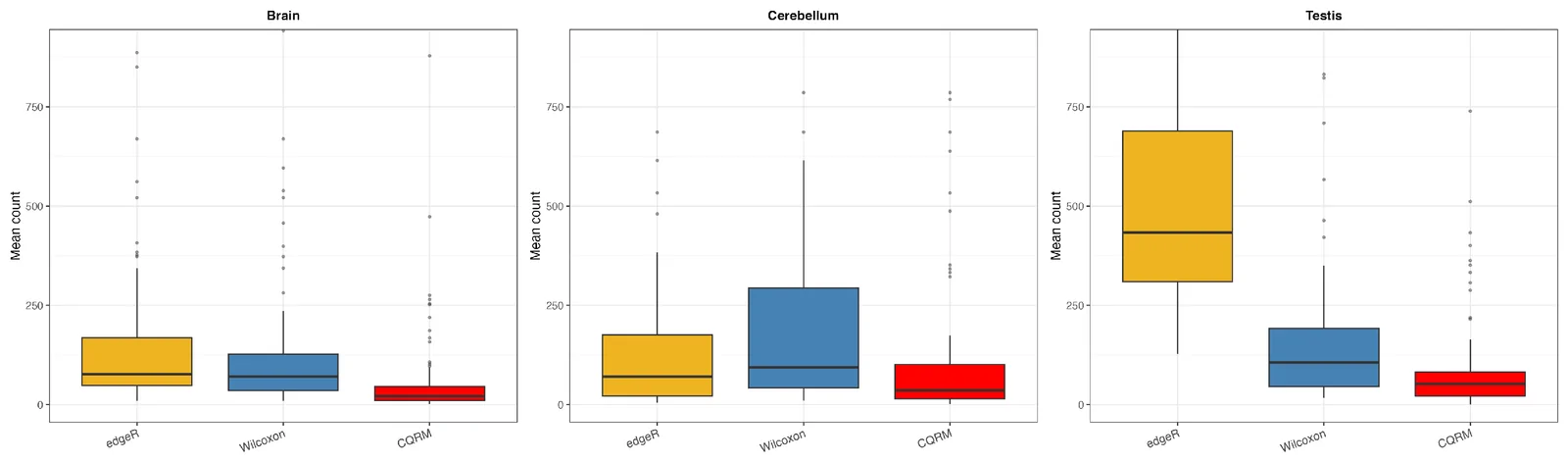
Case study 2: mean count of the 100 highest-ranked lncRNAs per method in each organ, ranked by each method’s raw *p*-value.

As shown in Figure 4, the CQRM identified a large core set of lncRNAs shared with edgeR and Wilcoxon across all three organs. Consistent with the simulation studies and Case Study 1, the lncRNAs prioritized by the CQRM tended to exhibit lower average expression levels than those identified by edgeR or Wilcoxon (Figure 5). To evaluate the functional relevance of the identified lncRNAs, we performed enrichment analysis for lncRNAs using the curated lncRNA resource NcPath [30], the results of which are included in Supplementary Tables S6–S8.

The top signaling pathways targeted by lncRNAs detected by the CQRM in the brain include those involved in neural metabolism and membrane formation (e.g., cholesterol metabolism, fat digestion and absorption, and phosphatidylinositol signaling), stem-cell and developmental regulation (e.g., pluripotency signaling and autophagy) and neuroimmune regulation (e.g., cytokine signaling and Th1/Th2 differentiation), all of which have established roles in brain development and maturation [31,32,33]. For cerebellum, the genes targeted by lncRNAs detected by the CQRM were mainly enriched in pathways involved in neural circuit formation and cerebellar maturation (e.g., axon guidance, Rap1 signaling pathway, and circadian rhythm). For testis, the genes targeted by lncRNAs detected by the CQRM were mainly enriched in pathways involved in endocrine and reproductive signaling (e.g., insulin secretion, thyroid hormone signaling pathway, and oxytocin signaling pathway). Notably, these enriched pathways are highly relevant to the biological functions and developmental processes of their respective organs, supporting the ability of the CQRM to identify biologically meaningful lncRNAs and their target genes. The distinct enrichment patterns observed across organs further highlight the tissue-specific regulatory roles of lncRNAs during development.

## 3. Discussion

In this paper, we developed the CQRM, a composite quantile regression framework for DE analysis of ncRNA count data. Unlike conventional DE methods that primarily target differences in mean expression, the CQRM aggregates information across multiple quantiles and detects a broader range of distributional changes, including shifts in variability, skewness, and multimodality. These features are commonly observed across diverse ncRNA species and often reflect biologically meaningful regulatory processes. The simulations showed that the CQRM improved DE gene detection power with overall better FDR control compared to other methods, especially for multimodal expression differences and low-count scenarios. The two real-data applications further support the practical utility of the CQRM. In both the broad-spectrum Smart-seq-total scRNA-seq dataset and the human organ developmental lncRNA sequencing dataset, the CQRM demonstrated increased sensitivity for low-abundance ncRNAs while retaining the majority of consensus differential expression signals that were identified across the methods. Moreover, for the organ developmental example, target genes of lncRNAs prioritized by the CQRM are enriched in pathways that are highly related to the developmental process of specific organs, suggesting that the CQRM detects biologically meaningful signals rather than artifacts of increased false-positive findings.

To date, the majority of DE methods have been developed for protein-coding genes and rely on distributional assumptions that are often well suited to mRNA expression profiles. Although several recent studies have focused on identifying critical ncRNA regulators and reconstructing ncRNA-mediated regulatory networks [34,35,36], less effort has been made to develop DE methods that are specifically tailored to the unique characteristics of ncRNA sequencing data. Many ncRNAs exhibit low abundance, high sparsity, high biological variability, and heterogeneous or multimodal expression distributions [9,20]. Consequently, DE may arise through changes in multiple aspects of the expression distribution rather than solely through shifts in mean expression. The CQRM addresses this gap by providing a flexible distribution-aware framework that aggregates evidence across multiple quantiles, which can improve statistical efficiency and robustness relative to single-quantile analyses, particularly under heterogeneous and non-Gaussian error distributions [37]. Although quantile regression has previously been applied for DE analysis in scRNA-seq data such as ZIAQ [18], the CQRM captures a broader spectrum of DE patterns without relying on explicit zero-inflation assumptions, thereby showing particular advantages for ncRNA expression data.

Extreme sparsity, such as in some single-cell RNA-seq data, limits the available information for quantile inference, and jittering does not eliminate this limitation. We have shown in simulations that, as zero inflation becomes more severe, gene recovery declines across methods, including the CQRM. These results support the CQRM’s utility under moderate sparsity while confirming that jittering alone cannot overcome the information loss caused by extreme sparsity. Calibration also depends on sample size and quantile selection; extreme quantiles and grids that are too narrow showed greater FDR inflation. We therefore recommend a prespecified interior grid, such as (0.2,0.4,0.6,0.8). Repeated quantile fitting makes the CQRM computationally demanding as sample size increases despite comparable memory requirements to those of competing methods. Gene-level parallelization and the approximation version of the CQRM can reduce runtime. The CQRM accommodates both bulk RNA-seq samples compared across conditions and individual cells compared across cell types or cell lines, but its current formulation does not account for correlation among cells from the same donor. For comparisons across biological conditions involving multiple donors, donor-level pseudobulk analysis can preserve biological replication and reduce computational demands. The current framework analyzes each ncRNA independently and therefore does not explicitly leverage regulatory relationships, co-expression patterns, or pathway structure that may further improve power and biological interpretability. Future work will focus on integrating prior biological knowledge, gene regulatory networks, and multi-omics data within the composite quantile framework to better prioritize critical ncRNA markers, characterize ncRNA-mediated regulatory mechanisms and improve biological interpretation. Methodological extensions will address extreme sparsity and cell–cell correlation in single-cell data, extend inference to paired and mixed-effects designs, and validate performance in multifactor experiments. Sharing information across genes may further improve efficiency in small-sample settings. An R package implementing the CQRM and the codes that are required to reproduce the analyses presented in this study are publicly available at: https://github.com/iamverywell/CQRM (accessed on 27 June 2025).

## 4. Methods

**Figure 6:**
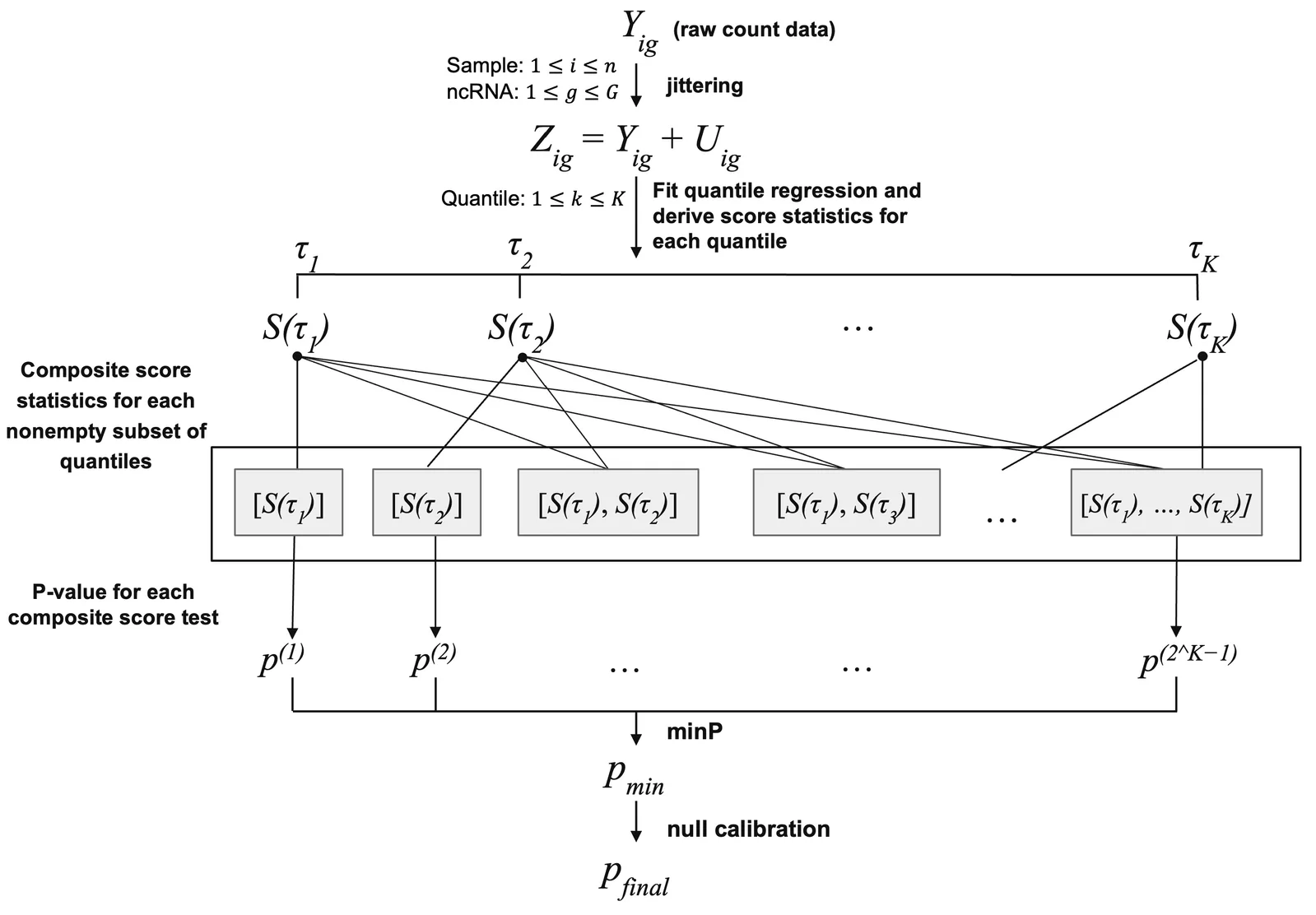
Overview of the CQRM procedure. Integer counts were first made continuous by jittering and transformation; the quantile regression was fitted to jittered data and the score statistic $S(\tau_{k})$ was derived for each level of the quantile grid $\left(\tau_{1},...,\tau_{K}\right)$; then the composite score statistic was derived for each non-empty subset of quantiles (a total of $2^{K}-1$ combinations) and tested using a $\chi^{2}$ statistic. The smallest resulting *p*-value is calibrated against a null distribution with the same covariance, which accounts for the multiplicity of the subset search.

We propose a composite quantile regression-based framework for differential expression (DE) analysis of ncRNA count data from RNA-seq experiments. The method first models the association between expression counts and covariates at individual quantiles using quantile regression with jittering to accommodate the discrete nature of count data. It then aggregates evidence for each non-empty subset of quantiles through a composite testing procedure and takes the subset with the minimum *p*-value, enabling the detection of differential expression patterns that may occur throughout the expression distribution rather than at a single quantile or solely in the mean. A detailed flowchart can be found in Figure 6.

### 4.1. Modeling ncRNA Count Data Using Quantile Regression with Jittering

Denote by **Y** the n×G matrix of count data from RNA-seq where $Y_{ig}$ indicates the count value for the *g*-th ncRNA (1≤g≤G) in sample *i* (1≤i≤n) from RNA-seq data. Denote by **X** the n×K matrix of covariates. In a typical DE analysis, we are interested in comparing the RNA expression profiles between a case and a control group. Without loss of generality, we assume one covariate (K=1), where $X_{i}=1$ when a sample is from the case group (e.g., cancer patients) and $X_{i}=0$ when a sample is from the control group (e.g., normal controls). Standard quantile regression (QR) assumes a continuous response variable. Directly applying QR to count data is challenging because the discreteness of the response leads to non-smoothness and non-standard asymptotic behavior of the conditional quantile estimator [38]. To address this issue, Machado and Santos Silva [39] proposed adding independent uniform noise to the count outcome, a procedure commonly referred to as jittering [40]. Specifically, let $U_{ig}$ denote the uniform random variable independent of $Y_{ig}$, and define the jittered response $Z_{ig}=Y_{ig}+U_{ig}$. The resulting variable $Z_{ig}$ is continuous, and its conditional quantiles have a one-to-one relationship with those of the original count variable under the jittering construction. After applying a monotonic transformation to the jittered count variable $T\left(Z_{ig}\right)=\log_{2}\left(Z_{ig}-\tau \right)$ if $Z_{ig}>\tau$ and $\log_{2}\left(\zeta \right)$ otherwise, where ζ is a suitably small positive number, the conditional quantile function of the transformed response can be modeled using quantile regression as

(1) $$Q_{T(Z_{ig})}\left(\tau |x_{i}\right)=\alpha_{g}\left(\tau \right)+x_{i}^{T}\beta_{g}\left(\tau \right),$$

where $Q_{T(Z_{ig})}\left(\tau |x_{i}\right)$ denotes the conditional τ-th quantile of $T(Z_{ig})$ given covariates $x_{i}$, $\alpha_{g}\left(\tau \right)$ is the quantile-specific intercept, and $\beta_{g}\left(\tau \right)$ is the vector of quantile-specific regression coefficients. The transformation $T(Z_{ig})$ we employed here is the same as in Machado and Santos Silva [39], which works as a reasonable approximation in many situations [39].

The coefficient estimates (α(τ),β(τ)) for any fixed τ can be obtained by

(2) $$\left({\hat{\alpha }}_{g}\left(\tau \right),{\hat{\beta }}_{g}\left(\tau \right)\right)=\underset{\alpha (\tau ),\beta (\tau )}{\mathrm{argmin}}\sum_{i=1}^{n}\rho_{\tau }\left(T\left(Z_{ig}\right)-\alpha_{g}-x_{i}^{T}\beta_{g}\left(\tau \right)\right),$$

where $\rho_{\tau }\left(u\right)=u\left\{\tau -I\left(u<0\right)\right\}$.

To decrease the estimate’s dependence on the random draw of $U_{ig}$, we averaged the QR estimates from *m* repeatedly and independently “jittered” samples and used the “average-jittering” estimator ${\hat{\beta }}_{g}^{A}\left(\tau \right)$ as our final estimate:

(3) $${\hat{\beta }}_{g}^{A}\left(\tau \right)=\frac{1}{m}\sum_{l=1}^{m}{\hat{\beta }}_{g}^{\left(l\right)}\left(\tau \right),$$

where ${\hat{\beta }}_{g}^{\left(l\right)}\left(\tau \right)$ is the estimate derived from the *l*-th jittered sample. The “average-jittering” estimator is more efficient than the estimate obtained with a single sample. Following Machado and Santos Silva [39], we also showed the asymptotic behavior of ${\hat{\beta }}_{g}^{A}\left(\tau \right)$ (see Appendix A).

Similarly, we derived $T^{A}\left(Z_{ig}\right)$ and ${\hat{\alpha }}^{A}$ as:

(4) $$\begin{array}{cc} T^{A}\left(Z_{ig}\right) & =\frac{1}{m}\sum_{l=1}^{m}T\left(Z_{ig}^{\left(l\right)}\right); \end{array}$$

(5) $$\begin{array}{cc} {\hat{\alpha }}_{g}^{A}\left(\tau \right) & =\frac{1}{m}\sum_{l=1}^{m}{\hat{\alpha }}_{g}^{\left(l\right)}\left(\tau \right). \end{array}$$

Properties of jittering:

- **Jittering preserves the count data information without assuming any count data distribution.** Adding $U_{ig}∼\mathrm{Unif}\left(0,1\right)$ produces a continuous response $Z_{ig}$ from which the original count is exactly recoverable as $\left\lfloor Z_{ig}\right\rfloor =Y_{ig}$ without assuming any count data distribution itself. This randomization resolves discreteness and ties, enabling quantile regression and supporting asymptotic inference under appropriate regularity conditions.
- **Jittering variance decreases in *m*.** From the asymptotic covariance of the average-jittering estimator (Appendix A),
  $$V^{A}\;=\;\frac{1}{m}\;D^{-1}AD^{-1}\;+\;\left(1-\frac{1}{m}\right)D^{-1}BD^{-1},$$
  the first term, which is the contribution of the random draw of *U*, decays as 1/m, while the second converges to the limiting covariance $D^{-1}BD^{-1}$ attained as m→∞. Increasing *m* therefore strictly leads to a reduction in variance. To balance computational cost and variance reduction, we chose m=50 for all implementations.
- **Robustness to different count distributions.** Jittering adds independent uniform noise to produce continuous data regardless of the original count data distribution, so it naturally accommodates overdispersed, zero-inflated, and multimodal counts without specifying a parametric count model. However, jittering alone does not guarantee valid inference; the subsequent quantile-based tests still require appropriate regularity conditions and sufficient sample sizes.

### 4.2. Composite Quantile Regression for Differential Expression Testing

A key limitation of existing QR-based differential expression methods is that inference is typically conducted at a single quantile level. Such approaches are most sensitive to differential expression occurring at that particular portion of the distribution but may lose power when expression differences are distributed across multiple quantiles or the affected quantiles vary across ncRNA species. Given the heterogeneous and often multimodal expression distributions observed in ncRNA sequencing data, differential expression may manifest through changes in location, variability, sparsity, or other distributional characteristics. Motivated by this observation, we propose a composite QR framework that aggregates evidence across multiple quantiles to capture a broader spectrum of DE patterns.

For a fixed quantile level τ, inference can be performed using a score test. Compared with asymptotic Wald-type inference, score tests avoid estimation of the conditional density function and generally exhibit improved finite-sample performance, particularly at extreme quantiles [41]. In contrast to bootstrap-based procedures, score tests are also computationally efficient, making them well suited for large-scale RNA-seq studies involving thousands of ncRNAs. For the quantile regression model introduced in Section 4.1, the hypothesis of interest at quantile τ (without loss of generality; we will drop subscript *g* from here on) is

(6) $$H_{0}:\beta \left(\tau \right)=0\;\mathrm{vs}.\;H_{1}:\beta \left(\tau \right)\neq 0.$$

The corresponding score statistic is

(7) $$S\left(\tau \right)=n^{-1/2}\sum_{i=1}^{n}\phi_{\tau }\left\{T^{A}\left(Z_{i}\right)-{\tilde{\alpha }}^{A}\left(\tau \right)\right\}x_{i}^{*},$$

where $\phi_{\tau }\left(u\right)=\tau -I\left\{u<0\right\}$ is the quantile score function, ${\tilde{\alpha }}^{A}\left(\tau \right)$ denotes the estimated intercept under the null, and $x_{i}^{*}$ is the *i*th element of the residualized covariate vector $X^{*}={\left(x_{1}^{*},\ldots ,x_{n}^{*}\right)}^{T}$. Specifically, $X^{*}=\left(I-P_{1}\right)X$, where $P_{1}$ is the projection matrix onto the space spanned by the intercept. Thus, under the one-covariate setting, $X^{*}$ is an n×1 vector representing the covariate after removing its projection onto the intercept space. S(τ) measures the association between the transformed expression $T^{A}\left(Z_{i}\right)$ and *X* at the τ-th conditional quantile, i.e., after removing the collinearity between *X* and the intercept via $X^{*}$. Rather than testing each quantile separately, our goal is to assess differential expression across multiple quantiles simultaneously. Let $\tau_{1},\tau_{2},\ldots ,\tau_{K}$ denote a prespecified set of quantile levels and let $\tau^{\left(w\right)}$ denote a specific non-empty subset of $\left\{\tau_{1},\tau_{2},\ldots ,\tau_{K}\right\}$ with up to $2^{K}-1$ possible subsets. The choice of quantiles involves a trade-off between capturing stronger signals and introducing additional noise. In practice, a tuning procedure is recommended to determine an appropriate set of quantiles that balances statistical power and Type I error control. In this study, we use the 0.2, 0.4, 0.6, and 0.8 quantiles for all CQRM-based methods. For any non-empty subset *w*, let $\tau^{\left(w\right)}={\left(\tau_{\left(1\right)},\tau_{\left(2\right)},\ldots ,\tau_{\left(h\right)}\right)}^{T}$ denote the corresponding quantile vector of size *h* and define the vector of rank-score statistics as $S^{\left(w\right)}={\left(S\left(\tau_{\left(1\right)}\right),S\left(\tau_{\left(2\right)}\right),\ldots ,S\left(\tau_{\left(h\right)}\right)\right)}^{T}$. We can then conduct a composite score test aggregating information across these quantiles. Under the null, $S^{\left(w\right)}$ follows an asymptotically multivariate normal distribution,

(8) $$S^{\left(w\right)}∼N\left(0,\Sigma^{\left(w\right)}\right),$$

where $\Sigma^{\left(w\right)}$ is the h×h covariance matrix whose $\left(j,j^{\prime }\right)$-th element is

(9) $$\sigma_{jj^{\prime }}=n^{-1}\left\{\min\left(\tau_{j},\tau_{j^{\prime }}\right)-\tau_{j}\tau_{j^{\prime }}\right\}X^{*T}X^{*},\;j,j^{\prime }=1,\ldots ,h.$$

In particular, when $j=j^{\prime }$, this reduces to the diagonal elements

(10) $$\sigma_{jj}=n^{-1}\tau_{j}\left(1-\tau_{j}\right)X^{*T}X^{*},\;j=1,\ldots ,h,$$

whereas, when $j\neq j^{\prime }$, the off-diagonal elements are

(11) $$\sigma_{jj^{\prime }}=n^{-1}\left\{\min\left(\tau_{j},\tau_{j^{\prime }}\right)-\tau_{j}\tau_{j^{\prime }}\right\}X^{*T}X^{*},\;j,j^{\prime }=1,\ldots ,h,\;j\neq j^{\prime }.$$

The composite rank score test statistic is then constructed based on $S^{\left(w\right)}$,

(12) $$R^{\left(w\right)}={S^{\left(w\right)}}^{T}{\Sigma^{\left(w\right)}}^{-1}S^{\left(w\right)},$$

which is shown to asymptotically follow a $\chi^{2}$ distribution with degrees of freedom equal to *h* under the null hypothesis. This statistic jointly evaluates differential expression across all the quantiles contained in the combination and therefore captures a wider range of distributional changes than single quantile tests.

To adaptively identify the most informative quantile combination for each ncRNA, we evaluate all $2^{K}-1$ non-empty quantile combinations and compute the corresponding *p*-values $p^{\left(w\right)}$. We then employ a minimum-*p*-value (minP) procedure [42] and treat the quantile combination with the minimum *p*-value as the one reflecting the significance of the coefficient in the hypothesis test for differential expression, i.e., $w_{best}=\underset{w}{\mathrm{argmin}}\;\;p^{\left(w\right)}$.

Since the minimum *p*-value $p^{\left(w_{best}\right)}$ does not follow a uniform null distribution, a conventional approach is to generate an empirical null distribution via permutation to compute an adjusted *p*-value. However, permutation is computationally expensive as it requires refitting the quantile regression model repeatedly for each gene. To address this, we approximate the null distribution using the asymptotic distribution of the composite score statistics. As in Equation (8), the composite score statistic asymptotically follows a multivariate Gaussian with a covariance matrix that is shared across all genes. We draw B samples from this null distribution and compute *p*-values for all $2^{K}-1$ combinations of quantiles using the corresponding submatrices of the covariance matrix. For each gene, the minimum *p*-value is then taken over all combinations with the same degrees of freedom as the observed best combination, forming a gene-specific empirical null distribution. The final adjusted *p*-value is computed as the proportion of null minimum *p*-values that are less than or equal to the observed minimum *p*-value.

Unlike conventional differential expression methods that primarily target mean shifts, the proposed composite quantile regression model (CQRM) framework aggregates evidence across multiple quantiles and adaptively identifies the quantile combination that best characterizes differential expression. As a result, CQRM is capable of detecting a broader range of distributional changes, including shifts affecting only the lower or upper tails, heterogeneous expression variability, and multimodal expression patterns commonly observed in ncRNA sequencing studies. To investigate alternative patterns of differential expression, we also developed two related procedures: CQRM-Region and CQRM-Best. CQRM-Region restricts the candidate quantile combinations to contiguous quantile intervals, thereby favoring differential expression signals that extend smoothly across neighboring portions of the expression distribution. In contrast, CQRM-Best considers all individual quantiles and selects the most significant quantile-specific test.

**Distinction from composite quantile regression (CQR) in Zou and Yuan [37].** Composite quantile regression (CQR), proposed by Zou and Yuan [37], is a classical composite quantile-based method that shares some similarity with our method. It estimates a common slope vector across quantiles, with quantile-specific intercepts, by minimizing a summed check loss. In contrast, CQRM fits separate quantile-specific regressions without a common-slope constraint, allowing covariate effects to vary across the expression distribution. Combination occurs at the inference stage: CQRM combines rank-score statistics over quantile subsets, accounts for their joint covariance, and calibrates the minimum *p*-value under their joint null distribution. Thus, CQRM is an adaptive multi-quantile hypothesis-testing framework, with “composite” referring to the combination of dependent evidence across quantiles.

**Distinction from ZIAQ [18].** Although CQRM and ZIAQ both fit quantile-specific regressions, they differ in two key respects. First, ZIAQ analyzes normalized continuous expression, whereas CQRM uses repeated jittering to perform quantile inference directly on raw counts, preserving their discrete, low-count, and zero-inflation structure. Second, ZIAQ combines quantile-specific *p*-values using Fisher’s method without accounting for their dependence. CQRM instead combines rank-score statistics using their joint covariance, evaluates candidate quantile subsets, and calibrates the minimum *p*-value under the joint multivariate null distribution.

**Bulk RNA-seq vs. single-cell RNA-seq.** When applying CQRM to bulk and single-cell RNA-seq data, both use a count matrix and a design matrix: in bulk data, observations are biological samples compared across conditions; in our single-cell application, observations are individual cells compared across cell types or cell lines. Single-cell data typically have greater sparsity, which can limit quantile inference, and cells from the same donor may be correlated, which the current formulation does not model. These distinctions and limitations are also described in Section 3.

**Biological interpretation of combining multiple quantiles.** Combining multiple quantiles allows CQRM to detect expression differences across the distribution: lower quantiles can reflect differences among low-expressing cells or samples, middle quantiles capture changes in typical expression, and upper quantiles reflect highly expressing subpopulations. Jointly evaluating these quantiles can reveal heterogeneous expression changes that may be missed by comparing means alone.

## 5. Conclusions

In this paper, we developed CQRM, a composite quantile regression method for DE analysis of ncRNA count data. By combining evidence across quantiles, CQRM improved detection of multimodal expression difference, complementing methods that primarily target changes in mean expression. Two real-data applications identified ncRNAs and associated pathways consistent with cell-type-specific biology and organ development, supporting the biological relevance of the findings. By capturing heterogeneous ncRNA expression changes, CQRM will facilitate the selection of candidate ncRNA biomarkers and the investigation of regulatory processes relevant to development and disease.

## Data Availability

The scRNA-seq dataset used in Case Study 1 is publicly available at NCBI GEO under accession number GSE151334 (accessed on 30 June 2025). The bulk RNA-seq dataset used in Case Study 2 is publicly available at LncExpDB (https://ngdc.cncb.ac.cn/lncexpdb/downloads; accession E-MTAB-6814; accessed on 27 June 2025).

## Supplementary Materials

The following supporting information can be downloaded at: https://www.mdpi.com/article/10.3390/ncrna12050039/s1, Figure S1: Representative lncRNA/ncRNA expression distributions illustrating similar group means but distinct distribution shapes from case study examples. Figure S2: Model-based simulation: proportion of all true DEG recovered among the top-ranked genes for all methods. Bimodal DE = 75%, Multi-modal DE = 25%. Figure S3: Model-based simulation: empirical against nominal FDR for nine methods. Bimodal DE = 75%, Multi-modal DE = 25%. Figure S4: (a) Empirical FDR of all methods at nominal FDR = 0.05 against zero inflation fraction; (b) The proportion of true DE genes among the top 400 genes detected by all methods against zero inflation fraction. Figure S5: Performance of CQRM over different quantile choices (left: power; right: FDR control). Figure S6: The impact of sample size on the performance of CQRM and other methods (left: power; right: FDR control). Figure S7: Computational benchmark. Figure S8: Case study 1, overlap of differentially expressed ncRNAs for the two RNA classes: snoRNA (a–c) and snRNA (d–f). Table S1: Theoretical computational complexity of CQRM variants and reference DE methods. Table S2: Peak session memory (MB) for each method. Table S3: Annotated top-ranked ncRNAs—Fibroblast vs. HEK293T comparison, Case study 1. Table S4: Annotated top-ranked ncRNAs—Fibroblast vs. MCF7 comparison, Case study 1. Table S5: Annotated top-ranked ncRNAs—HEK293T vs. MCF7 comparison, Case study 1. Table S6: NcPath/KEGG pathway enrichment for lncRNA target genes in Brain, Case study 2. Table S7: NcPath/KEGG pathway enrichment for lncRNA target genes in Cerebellum, Case study 2. Table S8: NcPath/KEGG pathway enrichment for lncRNA target genes in Testis, Case study 2.

## Appendix A

The asymptotic behavior of the average-jittering estimator ${\hat{\beta }}_{g}^{A}\left(\tau \right)$ defined in Equation (3) is as follows:

(A1) $$\sqrt{n}\left({\hat{\beta }}_{m}^{A}\left(\tau \right)-\beta \left(\tau \right)\right)\to^{D}N\left(0,V^{A}\right),$$

where

(A2) $$V^{A}=\frac{1}{m}D^{-1}AD^{-1}+\left(1-\frac{1}{m}\right)D^{-1}BD^{-1},$$

where $A=\tau \left(1-\tau \right)E\left(XX^{T}\right)$, $D=E(f_{T}\left(X^{T}\beta \left(\tau \right)|X\right)XX^{T})$, and

(A3) $$B=E\left\{\left[\tau \left(1-\tau \right)-\eta \left(X\right)\right]XX^{T}\right\}$$

and

(A4) $$\begin{array}{cc} \eta \left(X\right)=f_{Y|x}\left(Q_{Y}\left(\tau |x\right)\right)\{\left[Q_{Z}\left(\tau |x\right)-Q_{Y}\left(\tau |x\right)\right] & \;\\ \; & \;\times [Q_{Y}\left(\tau |x\right)+1-Q_{Z}\left(\tau |x\right)]\}. \end{array}$$

As the repetition (m) goes to infinity, the limiting covariance will be $D^{-1}BD^{-1}$ [39].
