# Supplementary material for: CQRM: A Composite Quantile Regression Model for Differential Expression Analysis of Non-Coding RNA Count Data
Source: Noncoding RNA. 2026 Oct 13;12(5):39. doi: 10.3390/ncrna12050039 (PMC13708692; doi:10.3390/ncrna12050039)

Supplementary Figures

Figure S1. Representative lncRNA/ncRNA expression distributions illustrating similar group means but distinct distribution shapes from case study examples. Panels (a)-(b): Case study 2 organ lncRNAs (Brain, Cerebellum); panels (c)-(d): Case study 1 scRNAseq ncRNAs (HEK293T vs. MCF7).

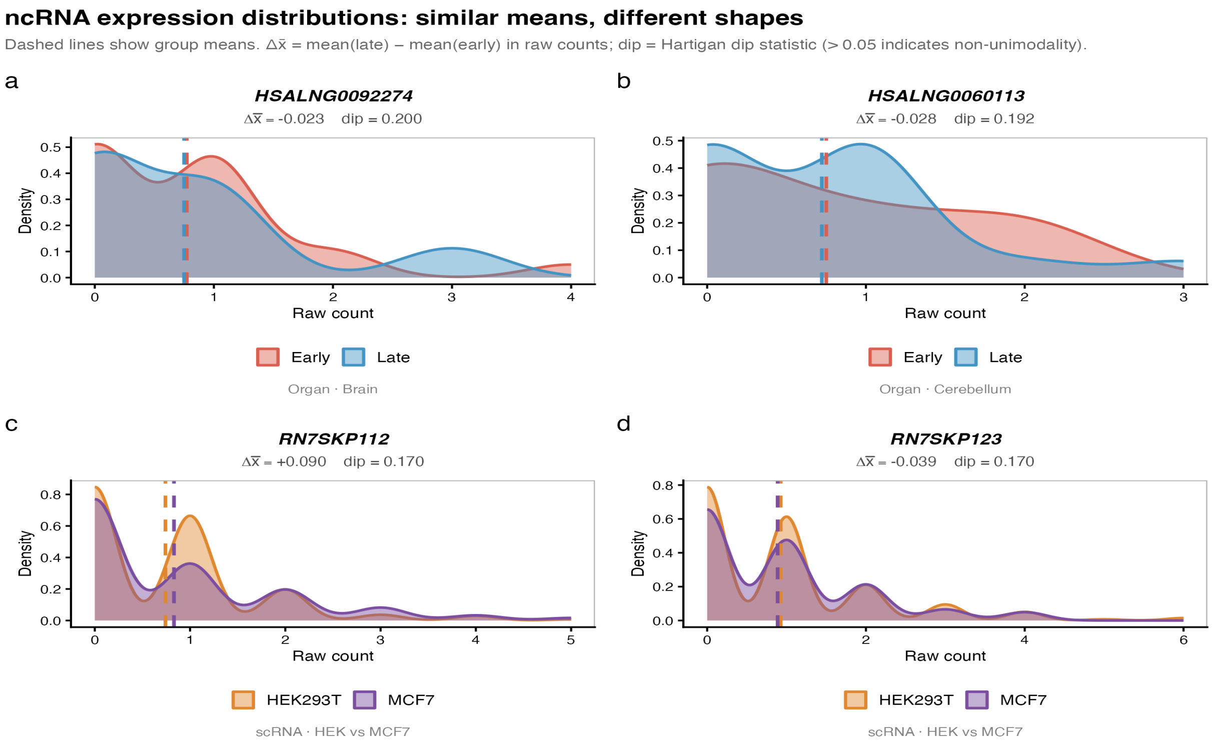

Figure S2. Model-based simulation: proportion of all true DEG recovered among the top-ranked genes for nine methods. Bimodal DE=75%, Multi-modal DE=25%. (a)-(c): moderate-count regime at the weakest, median and strongest signal levels. (d)-(f): low-count regime, same three levels.

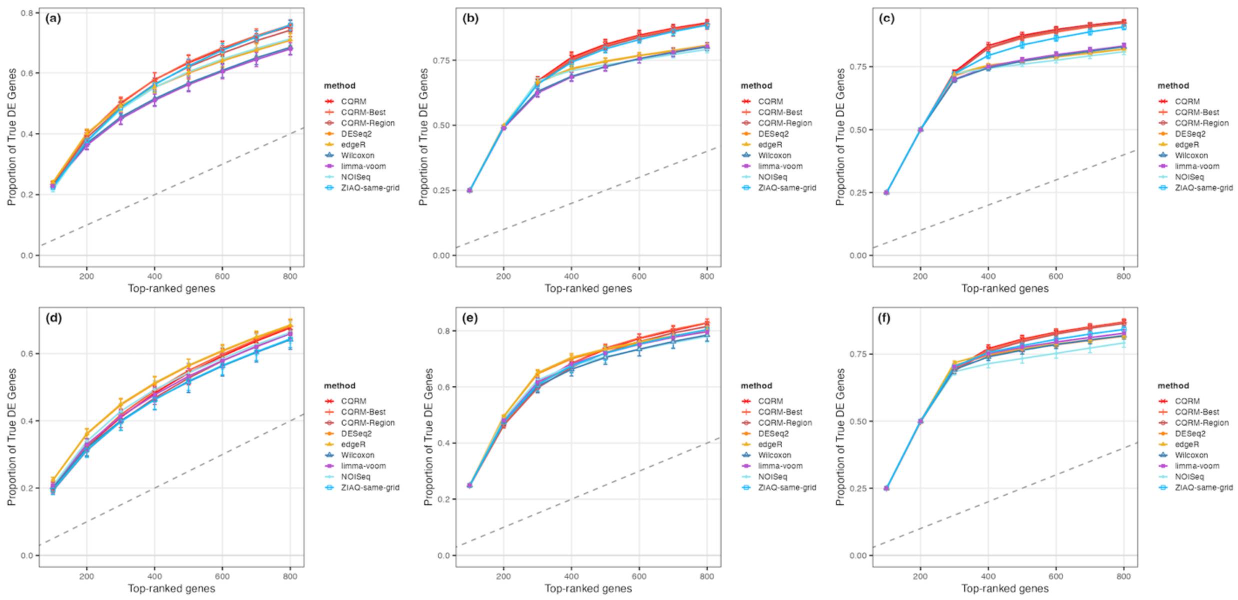

Figure S3. Model-based simulation: empirical against nominal FDR for nine methods. Bimodal DE=75%, Multi-modal DE=25%. (a)-(c): moderate-count regime at the weakest, median and strongest signal levels. (d)-(f): low-count regime, same three levels.

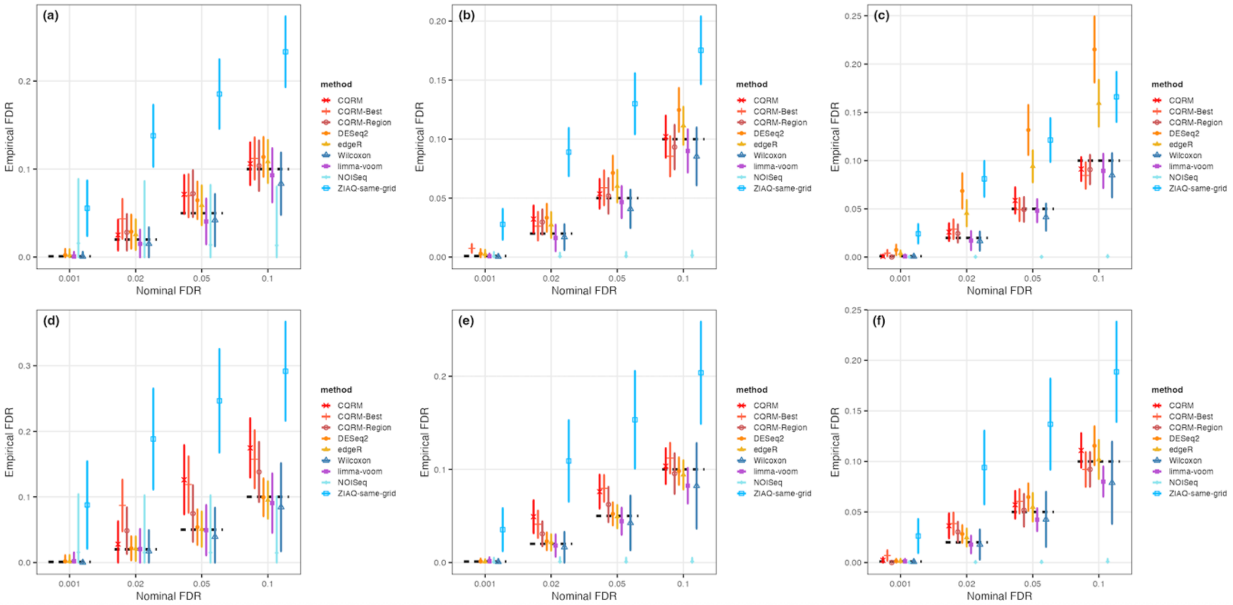

Figure S4. (a) Empirical FDR of all methods at nominal FDR=0.05 against zero inflation fraction; (b) The proportion of true DE genes among the top 400 genes detected by all methods against zero inflation fraction.

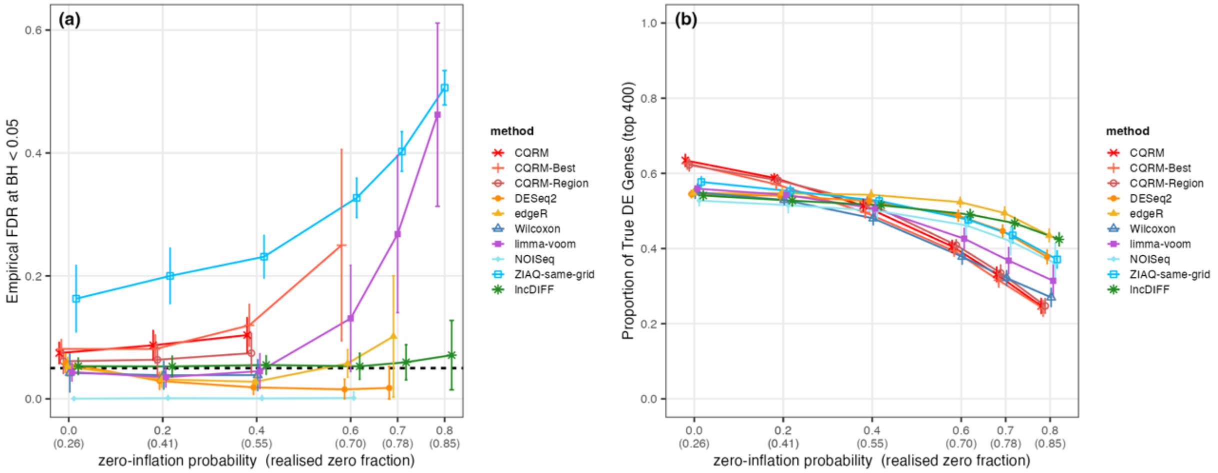

Figure S5. Performance of CQRM over different quantile choices (left: power; right: FDR control).

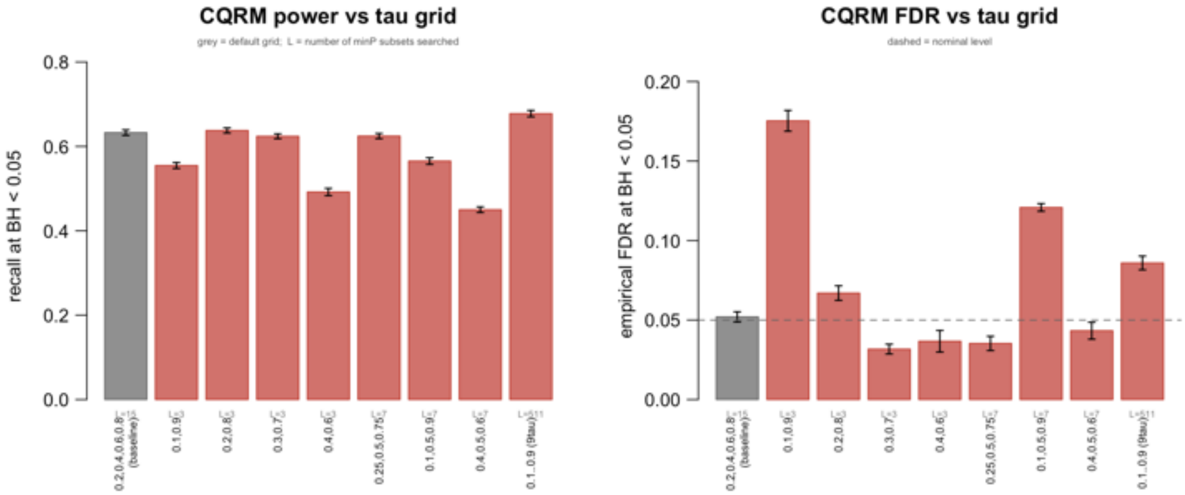

Figure S6. The impact of sample size on the performance of CQRM and other methods (left: power; right: FDR control)

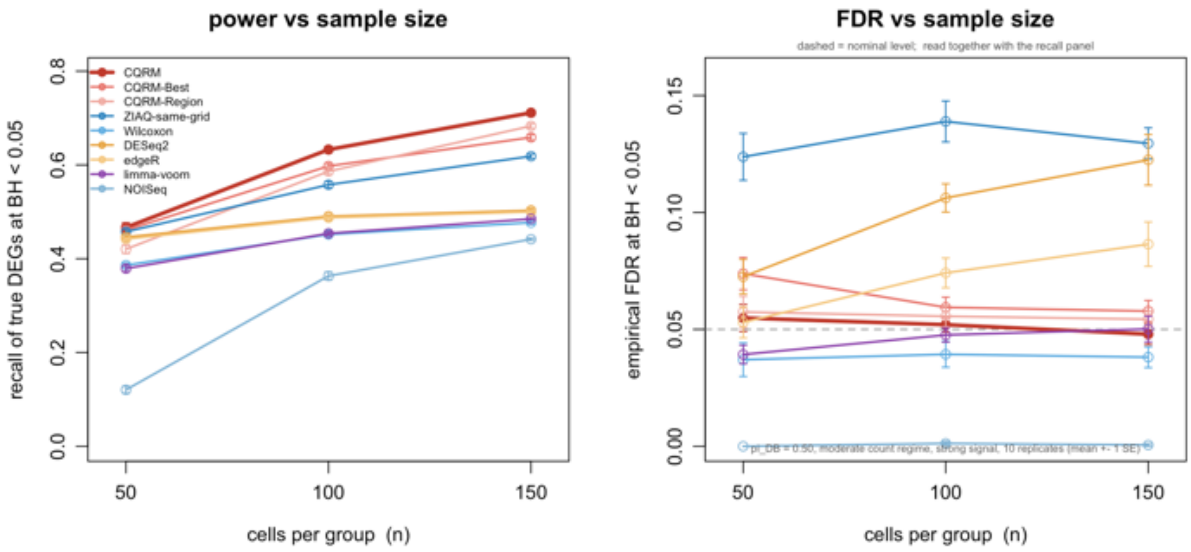

Figure S7. Computational benchmark. (a,b) Runtime and peak session memory at G=2,000 genes, n=50 per group, B=1,000, M=50. (c) Runtime against sample size at G=2,000 with varying sample sizes. Entries marked * were timed on 20 genes and scaled linearly: those methods test each gene independently, whereas the mean-based methods share information across genes and were run at the full G. All methods in (a) and (b) were measured in a single R session with every package attached before timing, since peak session memory depends on what is loaded. DESeq2 uses poscounts size factors. Panel (c) covers the seven methods run along the sample-size axis.

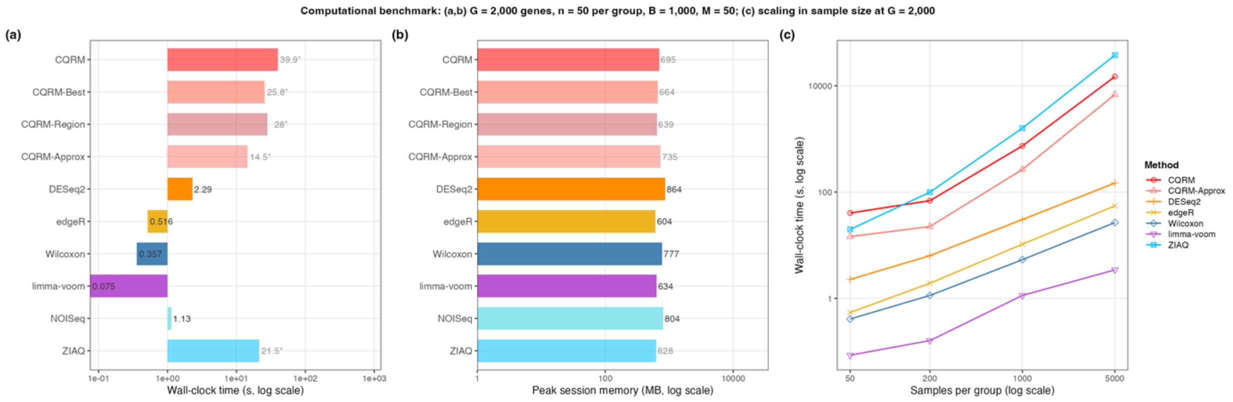

Figure S8. Case study 1, overlap of differentially expressed ncRNAs for the two RNA classes: snoRNA (a–c) and snRNA (d–f).

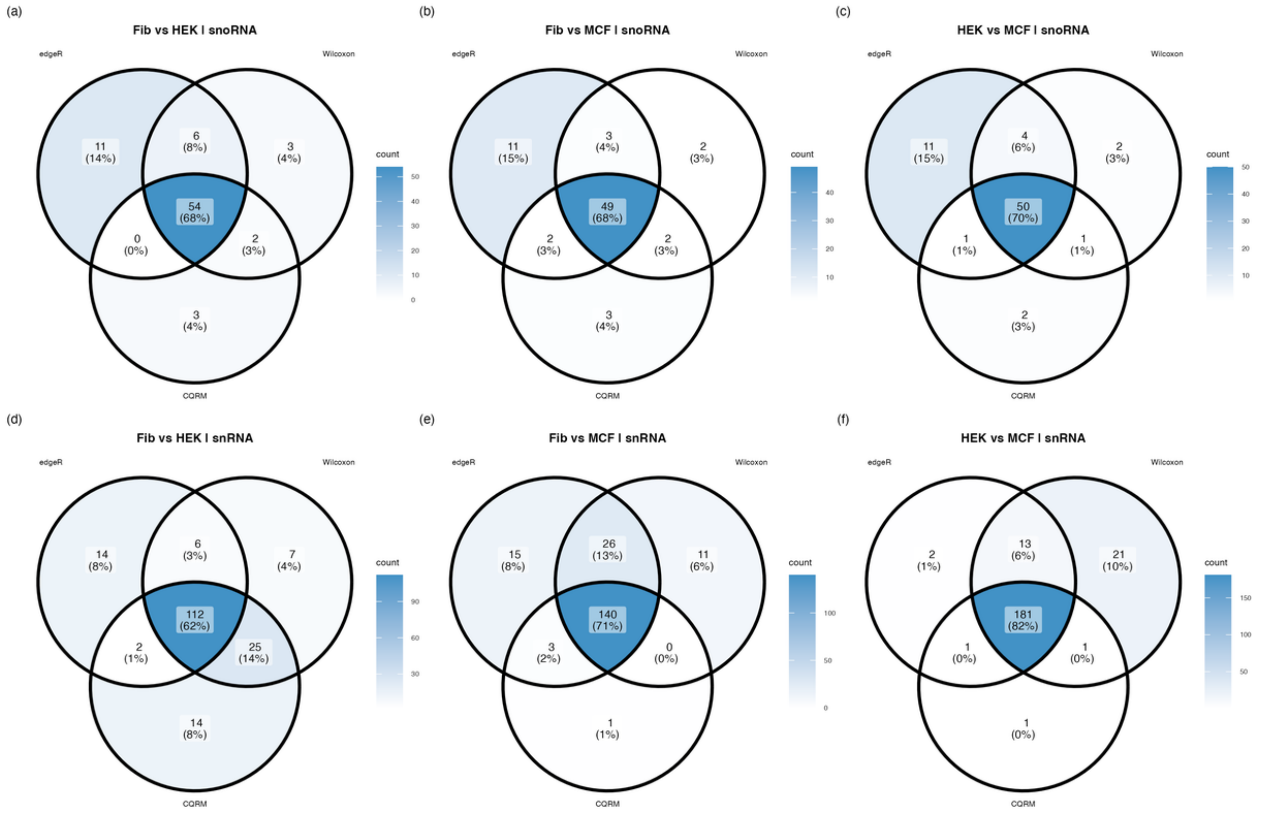

Supplement: Supplementary file 1 [file ncrna-12-00039-s001.zip › ncrna-4430434-supplementary-figures.pdf]
