# Supplementary material for: CQRM: A Composite Quantile Regression Model for Differential Expression Analysis of Non-Coding RNA Count Data
Source: Noncoding RNA. 2026 Oct 13;12(5):39. doi: 10.3390/ncrna12050039 (PMC13708692; doi:10.3390/ncrna12050039)
Supplement: Supplementary file 1 [file ncrna-12-00039-s001.zip › ncrna-4430434-supplementary-tables.pdf]

Table S1. Theoretical computational complexity of CQRM variants and reference DE methods.
G: genes; n: sample size; M: jittering replicates; k: quantile levels; B: permutation samples.

**Method**	**Computational Complexity**
CQRM	O(G x n x M x (2^k - 1 + B))
CQRM-Best	O(G x n x M x k + B)
CQRM-Region	O(G x n x M x (k - 1 + B))
CQRM-Approx	O(G x n x (2^k - 1))
DESeq2	O(G x n)
edgeR	O(G x n)
Wilcoxon	O(G x n x log n)
limma-voom	O(G x n)
NOISeq	O(G x n x r)
ZIAQ	O(G x n x k)

Table S2.Peak memory along the sample-size axis. Panel (a): n samples per group at G=2,000 genes. Panel (b): G genes at n=50 per group. Times in seconds, memory in MB, B=1,000, M=50, =(0.2,0.4,0.6,0.8). Entries marked * were timed on 20 genes and scaled linearly to the target G: CQRM, CQRM-Approx and ZIAQ test each gene independently, whereas the mean-based methods share informat

**Methods**	**n=50**	**200**	**1000**	**5000**
CQRM	693	666	733	1,779
CQRM-Approx	726	634	732	1,396
ZIAQ	605	587	640	1,214
DESeq2	779	752	1,246	3,526
edgeR	562	615	788	1,900
limma-voom	589	606	917	1,770
Wilcoxon	672	640	1,034	2,092

Table S3. Annotated top-ranked ncRNAs -- Fibroblast vs HEK293T comparison, Case study 1. The top 100 ncRNAs by CQRM's calibrated ranking (log-scale minimum p-value) were cross-referenced against LncBook v2.0, NCBI Gene, GeneCards and the primary literature, using HGNC as the authority for gene/locus identity. Columns: rank = position in CQRM's top-100 list; ncRNA = gene symbol; p_calibrated = Monte-Carlo calibrated CQRM p-value (floored at the resolution limit 1/(B+1) for many entries, hence the ties); log10_minp = log10 of the underlying minimum p-value on the continuous scale, which resolves ranking within tie groups; name_class = whether the symbol is an official HGNC name or a pseudogene/clone identifier; cluster = gene family/cluster membership where applicable (e.g. MIR, SCARNA, SNORD, RNU); hgnc_locus_type = HGNC locus-type annotation (e.g. RNA, long non-coding; RNA, small nuclear); hgnc_pseudogene = HGNC pseudogene flag; RefSeq = RefSeq accession if annotated; LncBook = LncBook v2.0 expression/functional annotation, or NA if absent; NCBI_Gene = NCBI Gene summary text, or NA; Literature = curated literature note, with [direct] marking a report specific to one of the three cell lines and [co-mention] a report that discusses the gene without a direct experimental link to this contrast. Only entries carrying information in at least one of the three resources are listed; entries with no external annotation (chiefly pseudogenes and clone-named loci) are omitted from this view. A numbered reference list (global numbering across all three contrasts) follows the table.

rank	ncRNA	p_calibratec	log10_minp	name_class	cluster	hgnc_locus_	hgnc_pseuc	RefSeq	LncBook	NCBI_Gene	Literature
2	RNU1-85P	0.000269	-195.35	pseudogene		RNA, small	FALSE	NG_04716(	NA	Predicted tc	NA
4	MIR4435-2HG	0.000269	-109.09	named-symbol		RNA, long r	FALSE	NR_01539(	Expr: norma	NA	[direct] brea
5	VTRNA1-1	0.000269	-102.28	named-symbol		RNA, vault	FALSE	NR_02670(	NA	Vaults are la	NA
6	MIR222	0.000269	-94.84	named-sym	MIR	RNA, micro	FALSE	NR_02963(	NA	microRNAs	NA
12	SCARNA23	0.000269	-79.91	named-sym	SCARNA	RNA, small	FALSE	NR_003007	NA	Predicted tc	NA
17	RN7SK	0.000269	-73.95	named-symbol		RNA, long r	FALSE	NR_00144(	NA	Predicted tc	[direct] HUM
18	SNORD114-11	0.000269	-72.43	named-sym	SNORD	RNA, small	FALSE	NR_003204	NA	Predicted tc	NA
19	REV3L-IT1	0.000269	-72.34	named-symbol		RNA, long r	FALSE	NA	Expr: norma	NA	single hit or
22	SNORD3A	0.000269	-68.48	named-sym	SNORD	RNA, small	FALSE	NR_00688(	NA	U3 RNA, ar	NA
24	RNU4-2	0.000269	-63.22	named-sym	RNU	RNA, small	FALSE	NR_003137	NA	Predicted tc	NA
28	SNORD7	0.000269	-58.36	named-sym	SNORD	RNA, small	FALSE	NR_003037	NA	This gene e	NA
29	JPX	0.000269	-58.3	named-symbol		RNA, long r	FALSE	NR_024582	Expr: norma	JPX is a nor	[co-mention
38	MIR23A	0.000269	-51.62	named-sym	MIR	RNA, micro	FALSE	NR_02949(	NA	microRNAs	NA
45	RNY3	0.000269	-39.94	named-symbol		RNA, Y	FALSE	NR_004392	NA	Predicted tc	NA
49	SCARNA14	0.000269	-39.06	named-sym	SCARNA	RNA, small	FALSE	NR_00438(	NA	Predicted tc	NA
50	SCARNA1	0.000269	-38.87	named-sym	SCARNA	RNA, small	FALSE	NR_002997	NA	Predicted tc	NA
51	LINC00998	0.000269	-38.21	named-symbol		gene with p	FALSE	NM_00135(	NA	Involved in	[co-mention
52	SNORA79	0.000269	-38.03	named-sym	SNORA	RNA, small	FALSE	NR_003021	NA	Predicted tc	NA
56	MIRLET7D	0.000269	-34.62	named-symbol		RNA, micro	FALSE	NR_029481	NA	microRNAs	NA
59	SCARNA10	0.000269	-33.38	named-sym	SCARNA	RNA, small	FALSE	NR_004387	NA	Predicted tc	NA
60	KCNQ1OT1	0.000269	-31.52	named-symbol		RNA, long r	FALSE	NR_00272(	Expr: norma	Human chrc	[direct] FIBR
61	SNORA59B	0.000269	-31.32	named-sym	SNORA	RNA, small	FALSE	NR_003022	NA	Predicted tc	NA
62	GLIDR	0.000269	-31.19	named-symbol		RNA, long r	FALSE	NR_01536(	Expr: norma	NA	NSCLC and
66	RNU6-1	0.000269	-30.45	named-sym	RNU	RNA, small	FALSE	NR_004394	NA	Predicted tc	NA
70	SNORA59A	0.000269	-29.23	named-sym	SNORA	RNA, small	FALSE	NR_00302(	NA	Predicted tc	NA
71	ATP2A1-AS1	0.000269	-29.04	named-symbol		RNA, long r	FALSE	NR_046287	Expr: norma	NA	cervical / co
74	RNU1-1	0.000269	-28.69	named-sym	RNU	RNA, small	FALSE	NR_00443(	NA	Predicted tc	NA
76	EPB41L4A-AS1	0.000269	-28.28	named-symbol		RNA, long r	FALSE	NR_01537(	Expr: norma	Predicted tc	[direct] brea
78	SNORD3C	0.000269	-28.15	named-sym	SNORD	RNA, small	FALSE	NR_006881	NA	Predicted tc	NA
81	SNORD1B	0.000269	-27.63	named-sym	SNORD	RNA, small	FALSE	NR_00439(	NA	Predicted tc	NA
83	MIR24-2	0.000269	-27.44	named-sym	MIR	RNA, micro	FALSE	NR_029497	NA	microRNAs	NA
85	RNU5F-1	0.000269	-26.42	named-sym	RNU	RNA, small	FALSE	NR_00275(	NA	Predicted tc	NA
88	RNU4-1	0.000269	-25.48	named-sym	RNU	RNA, small	FALSE	NR_00392(	NA	Predicted tc	NA
89	MALAT1	0.000269	-25.44	named-symbol		RNA, long r	FALSE	NR_00281(	Expr: norma	This gene p	[direct] brea
96	RNU1-2	0.000269	-23.67	named-sym	RNU	RNA, small	FALSE	NR_004427	NA	Predicted tc	NA
98	RN7SL1	0.000269	-23.64	named-sym	RN7SL	RNA, long r	FALSE	NR_00271(	NA	The signal r	[co-mention
99	SNORA12	0.000269	-23.61	named-sym	SNORA	RNA, small	FALSE	NR_002954	NA	Small nucle	NA

#REF

#REF REFERENCES (global numbering, see lit_refs_numbered.tsv)

#REF [3] Zhang R et al. MIR4435-2HG promotes breast cancer evolution through miR-205-5p/UBE2N axis and exosome-mediated macrophage M2-like p

#REF [4] Liang C, Gong Z LncRNA KCNQ1OT1 promotes hypertrophic scar progression by regulating fibroblast proliferation, migration, and ECM accumu

#REF [6] Abasi M, Ranjbari J, Ghanbarian H 7SK small nuclear RNA (Rn7SK) induces apoptosis through intrinsic and extrinsic pathways in human embryo

#REF [11] Cortes-Arias A et al. Deletion of the MALAT1 RNA 3' end promotes transcript decay and inhibits proliferation in gastric and breast cancer cells. I

#REF [13] Yang F, Lv S LncRNA EPB41L4A-AS1 Regulates Cell Proliferation, Apoptosis and Metastasis in Breast Cancer. Ann Clin Lab Sci 2022 (PMID 3

#REF [14] Tariq S et al. Genomic data mining reveals hub genes and lncRNAs as prognostic biomarkers in breast cancer. Sci Rep 2025 (PMID 41083591

#REF [15] Li Y et al. Context-dependent roles of lncRNA JPX in human cancers. Discov Oncol 2026 (PMID 42268347)

#REF [16] Abbaspour M et al. Immune Checkpoint Blockade and Emerging Combination Platforms in Breast Cancer: A Narrative Review. Breast Cancer (D

#REF [18] Chen X et al. The hidden players: LncRNA-Encoded micropeptides in cancer hallmarks. Cell Mol Life Sci 2026 (PMID 41961104)

Table S4. Annotated top-ranked ncRNAs -- Fibroblast vs MCF7 comparison, Case study 1. Same construction, columns and annotation sources as Table S3 (LncBook v2.0, NCBI Gene, GeneCards, literature, HGNC identity), applied to CQRM's top-100 ranked ncRNAs for this pairwise comparison. DSCAM-AS1 and LINC00052, which rank first and second here and in the HEK293T vs MCF7 comparison, are estrogen-receptor-regulated lncRNAs with reported roles in ER-positive breast cancer biology (see Literature column and main-text citations).

rank	ncRNA	p_calibratec	log10_minp	name_class	cluster	hgnc_locus_	hgnc_pseuc	RefSeq	LncBook	NCBI_Gene	Literature
1	DSCAM-AS	0.000353	-143.11	named-symbol		RNA, long r	FALSE	NR_038896	Expr: norma	NA	[direct] ER+
2	LINC00052	0.000353	-96.68	named-symbol		RNA, long r	FALSE	NR_026869	Expr: norma	Predicted tc	[direct] brea
3	SCARNA20	0.000353	-84.86	named-sym	SCARNA	RNA, small	FALSE	NR_002999	NA	Predicted tc	NA
4	FAM83H-AS	0.000353	-73.81	named-symbol		gene with p	FALSE	NM_001381	NA	NA	[co-mention
5	SNORD3C	0.000353	-59.5	named-sym	SNORD	RNA, small	FALSE	NR_006881	NA	Predicted tc	NA
6	VTRNA1-1	0.000353	-59.5	named-sym	VTRNA	RNA, vault	FALSE	NR_026703	NA	Vaults are la	NA
7	SNORD3B-	0.000353	-58.45	named-sym	SNORD	RNA, small	FALSE	NR_003271	NA	Predicted tc	NA
13	SNORD3B-	0.000353	-57.35	named-sym	SNORD	RNA, small	FALSE	NR_003924	NA	Predicted tc	NA
16	RNY3	0.000353	-56.26	named-sym	RNY	RNA, Y	FALSE	NR_004392	NA	Predicted tc	NA
17	SNORD3A	0.000353	-56.26	named-sym	SNORD	RNA, small	FALSE	NR_006880	NA	U3 RNA, ar	NA
18	RNY1	0.000353	-55.75	named-sym	RNY	RNA, Y	FALSE	NR_004391	NA	Y RNAs are	NA
23	MIR222	0.000353	-53.28	named-symbol		RNA, micro	FALSE	NR_029636	NA	microRNAs	NA
25	REV3L-IT1	0.000353	-49.89	named-symbol		RNA, long r	FALSE	NA	Expr: norma	NA	single hit or
30	RNU5E-1	0.000353	-47.18	named-sym	RNU	RNA, small	FALSE	NR_002754	NA	Predicted tc	NA
37	DACT3-AS	0.000353	-41.76	named-symbol		RNA, long r	FALSE	NR_040041	Expr: norma	NA	[direct] canc
42	SNHG25	0.000353	-41.32	named-symbol		RNA, long r	FALSE	NR_132278	Expr: norma	Predicted tc	AML and co
43	RNU6ATAC	0.000353	-40.98	named-symbol		RNA, small	FALSE	NR_023344	NA	Predicted tc	NA
46	RNU5D-1	0.000353	-39.68	named-sym	RNU	RNA, small	FALSE	NR_002755	NA	Predicted tc	NA
50	SNORD114	0.000353	-38.12	named-sym	SNORD	RNA, small	FALSE	NR_003204	NA	Predicted tc	NA
63	SNORD104	0.000353	-34.91	named-sym	SNORD	RNA, small	FALSE	NR_004380	NA	Predicted tc	NA
65	SCARNA23	0.000353	-34.04	named-sym	SCARNA	RNA, small	FALSE	NR_003007	NA	Predicted tc	NA
74	RNVU1-19	0.000353	-32.69	named-sym	RNVU	RNA, small	FALSE	NR_104086	NA	Predicted tc	NA
75	RN7SK	0.000353	-32.4	named-symbol		RNA, long r	FALSE	NR_001445	NA	Predicted tc	[direct] HUM
76	RNU1-1	0.000353	-32.4	named-sym	RNU	RNA, small	FALSE	NR_004430	NA	Predicted tc	NA
79	MIR3609	0.000353	-30.51	named-symbol		RNA, micro	FALSE	NR_037403	NA	microRNAs	NA
80	RNVU1-18	0.000353	-30.44	named-sym	RNVU	RNA, small	FALSE	NR_004400	NA	Predicted tc	NA
82	GATA3-AS1	0.000353	-30.37	named-symbol		RNA, long r	FALSE	NR_104327	Expr: norma	NA	[direct] brea
84	RNVU1-7	0.000353	-30.02	named-sym	RNVU	RNA, small	FALSE	NR_004426	NA	Predicted tc	NA
86	KCNQ1OT1	0.000353	-29.57	named-symbol		RNA, long r	FALSE	NR_002728	Expr: norma	Human chro	[direct] FIBF
89	TBX2-AS1	0.000353	-29.34	named-symbol		RNA, long r	FALSE	NR_125749	Expr: norma	NA	[direct] tamo
94	RNU1-2	0.000353	-27.74	named-sym	RNU	RNA, small	FALSE	NR_004427	NA	Predicted tc	NA
95	MALAT1	0.000353	-26.99	named-symbol		RNA, long r	FALSE	NR_002819	Expr: norma	This gene p	[direct] brea
96	SCARNA21	0.000353	-26.97	named-sym	SCARNA	RNA, small	FALSE	NR_003000	NA	Predicted tc	NA
97	RNU5B-1	0.000353	-26.72	named-sym	RNU	RNA, small	FALSE	NR_002757	NA	Predicted tc	NA

#REF
#REF REFERENCES (global numbering, see lit_refs_numbered.tsv)

#REF [1] Niknafs YS et al. The lncRNA landscape of breast cancer reveals a role for DSCAM-AS1 in breast cancer progression. Nat Commu
#REF [2] Sanchez-Lopez JM et al. Decoding LINC00052 role in breast cancer by bioinformatic and experimental analyses. RNA Biol 2024 (F
#REF [4] Liang C, Gong Z LncRNA KCNQ1OT1 promotes hypertrophic scar progression by regulating fibroblast proliferation, migration, and
#REF [5] Chen Y et al. Cancer-associated fibroblasts: enablers of tumor drug resistance. J Transl Med 2026 (PMID 41580863)
#REF [6] Abasi M, Ranjbari J, Ghanbarian H 7SK small nuclear RNA (Rn7SK) induces apoptosis through intrinsic and extrinsic pathways in h
#REF [7] Tong X, Zhao Y, Huang P LncRNA GATA3-AS1-mediated miR-2116-5p interaction modulates TCF12 and contributes to podocyte dy
#REF [8] Yuan J, Yuan G SEMA3G, downregulated by ncRNAs, correlates with favorable prognosis and tumor immune infiltration in kidney re
#REF [11] Cortes-Arias A et al. Deletion of the MALAT1 RNA 3' end promotes transcript decay and inhibits proliferation in gastric and breast c
#REF [17] Muralidharan H et al. Breast Cancer Stem Cells Upregulate IRF6 in Stromal Fibroblasts to Induce Stromagenesis. Cells 2024 (PM

Table S5. Annotated top-ranked ncRNAs -- HEK293T vs MCF7 comparison, Case study 1. Same construction, columns and annotation sources as Table S3, applied to CQRM's top-100 ranked ncRNAs for this pairwise comparison.

rank	ncRNA	p_calibratec	log10_minp	name_class	cluster	hgnc_locus_	hgnc_pseuc	RefSeq	LncBook	NCBI_Gene	Literature
1	DSCAM-AS	0.000384	-138.15	named-symbol		RNA, long r	FALSE	NR_038896	Expr: norma	NA	[direct] ER+
2	LINC00052	0.000384	-93.38	named-symbol		RNA, long r	FALSE	NR_026869	Expr: norma	Predicted tc	[direct] brea
3	SCARNA20	0.000384	-69.82	named-symbol		RNA, small	FALSE	NR_002999	NA	Predicted tc	NA
4	MIR4435-2I	0.000384	-63.7	named-symbol		RNA, long r	FALSE	NR_015395	Expr: norma	NA	[direct] brea
6	SNORD3A	0.000384	-57.83	named-sym	SNORD	RNA, small	FALSE	NR_006880	NA	U3 RNA, ar	NA
7	SNORD3B-	0.000384	-57.83	named-sym	SNORD	RNA, small	FALSE	NR_003271	NA	Predicted tc	NA
8	SNORD3C	0.000384	-57.83	named-sym	SNORD	RNA, small	FALSE	NR_006881	NA	Predicted tc	NA
10	FAM83H-AS	0.000384	-57.68	named-symbol		gene with p	FALSE	NM_001381	NA	NA	[co-mention
11	SNORD3B-	0.000384	-57.25	named-sym	SNORD	RNA, small	FALSE	NR_003924	NA	Predicted tc	NA
13	RNY1	0.000384	-56.25	named-sym	RNY	RNA, Y	FALSE	NR_004391	NA	Y RNAs are	NA
19	RNY3	0.000384	-51.63	named-sym	RNY	RNA, Y	FALSE	NR_004392	NA	Predicted tc	NA
36	MALAT1	0.000384	-41.63	named-symbol		RNA, long r	FALSE	NR_002819	Expr: norma	This gene p	[direct] brea
39	RNU5E-1	0.000384	-40.17	named-sym	RNU	RNA, small	FALSE	NR_002754	NA	Predicted tc	NA
40	REV3L-IT1	0.000384	-40.01	named-symbol		RNA, long r	FALSE	NA	Expr: norma	NA	single hit or
41	RNU1-85P	0.000384	-39.68	pseudogene		RNA, small	FALSE	NG_047169	NA	Predicted tc	NA
47	SNORD7	0.000384	-37.78	named-sym	SNORD	RNA, small	FALSE	NR_003037	NA	This gene e	NA
49	ZFAS1	0.000384	-37.65	named-symbol		RNA, long r	FALSE	NR_003604	Expr: norma	This gene re	[direct] brea
50	RNU6-1	0.000384	-37.65	named-sym	RNU	RNA, small	FALSE	NR_004394	NA	Predicted tc	NA
52	RNU5F-1	0.000384	-36.63	named-sym	RNU	RNA, small	FALSE	NR_002753	NA	Predicted tc	NA
60	SNHG25	0.000384	-32.43	named-symbol		RNA, long r	FALSE	NR_132278	Expr: norma	Predicted tc	AML and cc
62	RNU6-9	0.000384	-31.42	named-sym	RNU	RNA, small	FALSE	NR_104080	NA	Predicted tc	NA
64	VTRNA1-1	0.000384	-30.37	named-sym	VTRNA	RNA, vault	FALSE	NR_026703	NA	Vaults are la	NA
72	DACT3-AS	0.000384	-28.68	named-symbol		RNA, long r	FALSE	NR_040041	Expr: norma	NA	[direct] canc
73	RNVU1-19	0.000384	-28.07	named-symbol		RNA, small	FALSE	NR_104086	NA	Predicted tc	NA
74	SNORD104	0.000384	-27.93	named-sym	SNORD	RNA, small	FALSE	NR_004380	NA	Predicted tc	NA
76	GATA3-AS1	0.000384	-27.75	named-symbol		RNA, long r	FALSE	NR_104327	Expr: norma	NA	[direct] brea
85	SNORD71	0.000384	-25.02	named-sym	SNORD	RNA, small	FALSE	NR_003059	NA	Predicted tc	NA
97	PVT1	0.000384	-22.45	named-symbol		RNA, long r	FALSE	NR_003367	Expr: norma	This gene re	[direct] brea

#REF

#REF REFERENCES (global numbering, see lit_refs_numbered.tsv)

#REF [1] Niknafs YS et al. The lncRNA landscape of breast cancer reveals a role for DSCAM-AS1 in breast cancer progression. Nat Commu

#REF [2] Sanchez-Lopez JM et al. Decoding LINC00052 role in breast cancer by bioinformatic and experimental analyses. RNA Biol 2024 (F

#REF [3] Zhang R et al. MIR4435-2HG promotes breast cancer evolution through miR-205-5p/UBE2N axis and exosome-mediated macropha

#REF [5] Chen Y et al. Cancer-associated fibroblasts: enablers of tumor drug resistance. J Transl Med 2026 (PMID 41580863)

#REF [7] Tong X, Zhao Y, Huang P LncRNA GATA3-AS1-mediated miR-2116-5p interaction modulates TCF12 and contributes to podocyte dy

#REF [9] Zhang X et al. LncRNA PVT1 drives macrophage polarization and triple-negative breast cancer progression via PPARŒ≥-mediated

#REF [10] Melone V et al. Identification of LncRNAs scaffolding a chromatin-associated gene regulatory complex in breast cancer cells comp

#REF [11] Cortes-Arias A et al. Deletion of the MALAT1 RNA 3' end promotes transcript decay and inhibits proliferation in gastric and breast c

#REF [12] Moeini R, AhsanAlkhat A, Ghorbian S Exploring the Role of Long Noncoding RNAs in Breast Cancer Progression: A Comprehensiv

#REF [17] Muralidharan H et al. Breast Cancer Stem Cells Upregulate IRF6 in Stromal Fibroblasts to Induce Stromagenesis. Cells 2024 (PM

Table S6. NcPath/KEGG pathway enrichment for lncRNA target genes in Brain (Case study 2, organ development, early vs late stage). adj.p = Benjamini-Hochberg adjusted p-value of the enrichment test for each method's called lncRNA set; n = number of matched lncRNAs contributing to that enrichment. Bold green rows mark pathways significant (adj.p < 0.05) for CQRM but not for edgeR or Wilcoxon (CQRM-exclusive). Only pathways with adj.p < 0.05 in at least one method are shown. Top signaling pathways for CQRM-detected lncRNAs in brain span neural metabolism and membrane formation (cholesterol metabolism, fat digestion and absorption, phosphatidylinositol signaling), stem-cell/developmental regulation (pluripotency signaling, autophagy) and neuroimmune regulation (cytokine signaling, Th1/Th2 differentiation), consistent with established roles in brain development and maturation.

Pathway	CQRM adj.p	CQRM n	edgeR adj.p	edgeR n	Wilcoxon adj.p	Wilcoxon n
Cholesterol metabolism	2.86e-10	34	0.787	8	7.29e-04	19
Viral protein interaction with cytokine and cytokine receptor	5.08e-10	27	0.787	5	0.001	14
Fat digestion and absorption	5.08e-10	29	0.923	3	0.025	11
Inflammatory bowel disease	6.21e-10	35	0.787	11	8.63e-04	20
Phosphatidylinositol signaling system	1.08e-09	36	0.833	8	8.63e-04	21
Th1 and Th2 cell differentiation	1.08e-09	35	0.787	9	0.047	14
Thyroid cancer	1.08e-09	30	0.787	8	0.014	13
Signaling pathways regulating pluripotency of stem cells	1.15e-09	38	0.847	9	0.049	16
Ferroptosis	1.15e-09	38	0.781	14	7.29e-04	24
Lipid and atherosclerosis	1.33e-09	42	0.787	14	0.014	21
Hepatocellular carcinoma	1.77e-09	42	0.787	13	0.002	24
Tuberculosis	1.77e-09	41	0.781	16	0.001	24
Estrogen signaling pathway	1.77e-09	39	0.787	12	0.049	17
Chemical carcinogenesis - DNA adducts	1.77e-09	29	0.787	8	0.005	14
Autophagy - animal	2.28e-09	37	0.787	10	0.014	18
Toxoplasmosis	2.28e-09	38	0.822	10	0.006	20
PD-L1 expression and PD-1 checkpoint pathway in cancer	**2.28e-09**	**34**	0.781	12	0.080	13
Hematopoietic cell lineage	2.28e-09	34	0.822	8	0.007	17
Insulin resistance	**2.28e-09**	**39**	0.787	12	0.054	17
Bile secretion	2.28e-09	32	0.787	9	0.006	16
B cell receptor signaling pathway	2.47e-09	35	0.781	13	0.006	18
Th17 cell differentiation	2.47e-09	34	0.787	10	0.029	15
T cell receptor signaling pathway	2.49e-09	36	0.781	14	0.019	17
Peroxisome	2.49e-09	34	0.781	12	0.002	19
C-type lectin receptor signaling pathway	2.49e-09	37	0.794	10	0.015	18
Transcriptional misregulation in cancer	**2.49e-09**	**46**	0.622	23	0.066	22
Parathyroid hormone synthesis, secretion and action	**2.49e-09**	**35**	0.787	10	0.071	14
Lysosome biogenesis	2.70e-09	32	0.822	7	0.014	15
Fluid shear stress and atherosclerosis	3.32e-09	36	0.787	11	0.021	17
Vitamin digestion and absorption	**5.45e-09**	**23**	0.787	4	0.053	8
Basal cell carcinoma	6.46e-09	28	0.847	5	0.013	13
Pancreatic secretion	7.77e-09	36	0.790	10	0.004	20
Bladder cancer	7.77e-09	31	0.908	5	7.29e-04	20
RIG-I-like receptor signaling pathway	9.75e-09	36	0.781	14	0.001	22
Hepatitis B	9.75e-09	41	0.781	17	8.73e-04	26
Amphetamine addiction	**9.75e-09**	**30**	0.787	9	0.056	12
Fc gamma R-mediated phagosome formation	1.11e-08	35	0.787	10	0.005	19
Salivary secretion	1.23e-08	35	0.870	8	0.012	18
Hedgehog signaling pathway	**1.26e-08**	**27**	0.787	6	0.070	10
PPAR signaling pathway	1.26e-08	37	0.870	9	0.004	21
Influenza A	1.61e-08	40	0.781	18	0.005	23
Natural killer cell mediated cytotoxicity	1.61e-08	35	0.787	13	0.003	20
TGF-beta signaling pathway	**1.76e-08**	**31**	0.787	8	0.090	12
Diabetic cardiomyopathy	1.76e-08	42	0.781	18	0.013	23
Mitophagy - animal	1.80e-08	29	0.833	6	0.003	16
Prostate cancer	2.26e-08	37	0.787	12	0.001	23
AMPK signaling pathway	2.28e-08	41	0.787	17	0.015	22
Epithelial cell signaling in Helicobacter pylori infection	**2.53e-08**	**28**	0.787	7	0.064	11
Insulin signaling pathway	2.61e-08	38	0.787	13	0.008	21
ECM-receptor interaction	2.61e-08	35	0.882	8	0.047	16
Pancreatic cancer	2.61e-08	36	0.787	12	8.73e-04	23
Fc epsilon RI signaling pathway	2.61e-08	33	0.787	9	0.013	17
Adherens junction	**2.62e-08**	**29**	0.787	8	0.270	9
Taste transduction	2.66e-08	32	0.781	12	7.29e-04	21
Gastric cancer	2.77e-08	38	0.787	13	0.002	23
p53 signaling pathway	2.95e-08	37	0.735	17	0.001	23
Type II diabetes mellitus	3.00e-08	30	0.787	8	0.013	15
Mineral absorption	**3.10e-08**	**28**	0.973	3	0.217	9
FoxO signaling pathway	3.19e-08	36	0.822	10	0.043	17
Renin-angiotensin system	3.19e-08	20	0.787	4	0.013	9
Osteoclast differentiation	3.28e-08	36	0.787	11	0.025	18
Glucagon signaling pathway	3.28e-08	35	0.781	14	0.015	18
Endocrine resistance	3.28e-08	37	0.787	12	0.003	22
Leishmaniasis	3.36e-08	29	0.787	8	0.009	15
Endocytosis	3.48e-08	40	0.883	11	0.023	21
Adipocytokine signaling pathway	**3.48e-08**	**38**	0.787	13	0.053	18
mTOR signaling pathway	3.57e-08	37	0.851	10	0.003	22
Circadian rhythm	3.85e-08	26	0.622	11	0.039	11
Neurotrophin signaling pathway	4.02e-08	34	0.822	9	0.006	19
Phototransduction	**4.29e-08**	**19**	0.878	2	0.090	6
Inflammatory mediator regulation of TRP channels	4.29e-08	30	0.922	5	0.014	15
Synaptic vesicle cycle	4.74e-08	28	0.973	3	0.013	14
Thyroid hormone signaling pathway	4.80e-08	36	0.787	11	0.048	17
JAK-STAT signaling pathway	4.83e-08	36	0.787	12	0.008	20
Kaposi sarcoma-associated herpesvirus infection	4.83e-08	41	0.787	17	0.013	23
Spinocerebellar ataxia	5.14e-08	36	0.833	10	0.049	17
Coronavirus disease	5.48e-08	37	0.781	16	0.014	20
Chronic myeloid leukemia	6.14e-08	36	0.793	11	8.63e-04	24
Proteoglycans in cancer	6.17e-08	37	0.787	13	0.044	18
Notch signaling pathway	6.32e-08	29	0.958	4	0.005	16
Colorectal cancer	6.36e-08	32	0.622	15	0.021	16
NOD-like receptor signaling pathway	6.47e-08	40	0.477	22	0.001	26
Measles	7.24e-08	37	0.781	16	0.014	20
Long-term potentiation	7.24e-08	28	0.787	8	0.029	13
TNF signaling pathway	8.29e-08	38	0.787	15	0.013	21
Glioma	8.33e-08	35	0.787	11	0.002	22
Human immunodeficiency virus 1 infection	8.37e-08	40	0.787	16	0.031	21
Epstein-Barr virus infection	8.46e-08	40	0.787	14	0.005	24
Cell cycle	8.81e-08	35	0.869	9	8.80e-04	23
GnRH secretion	**9.05e-08**	**32**	0.787	10	0.070	14
GnRH signaling pathway	**9.30e-08**	**31**	0.822	8	0.085	13
Breast cancer	9.32e-08	37	0.787	12	0.001	24
Neutrophil extracellular trap formation	9.48e-08	37	0.787	13	0.030	19
Cellular senescence	9.48e-08	38	0.787	13	7.29e-04	26
Vibrio cholerae infection	1.00e-07	27	0.787	7	0.005	15
HIF-1 signaling pathway	1.00e-07	34	0.787	13	0.030	17
Malaria	**1.02e-07**	**34**	0.885	8	0.228	13
Gastric acid secretion	1.15e-07	30	0.787	11	0.001	19
Central carbon metabolism in cancer	1.25e-07	32	0.787	12	0.014	17
Melanoma	1.26e-07	34	0.885	8	0.002	21
Chemical carcinogenesis - receptor activation	1.28e-07	40	0.781	18	0.003	25
Pathways in cancer	1.42e-07	45	0.787	20	0.005	28
Rap1 signaling pathway	1.44e-07	38	0.787	13	0.029	20
Cortisol synthesis and secretion	**1.55e-07**	**28**	0.787	8	0.064	12
Renal cell carcinoma	**1.55e-07**	**30**	0.851	7	0.071	13
Proximal tubule bicarbonate reclamation	**1.55e-07**	**20**	0.787	4	0.087	7
Viral carcinogenesis	1.61e-07	42	0.787	19	0.009	25
Apelin signaling pathway	2.02e-07	36	0.787	14	0.044	18
ErbB signaling pathway	**2.17e-07**	**31**	0.787	11	0.064	14
Hepatitis C	2.22e-07	39	0.787	15	0.002	25
Antifolate resistance	**2.28e-07**	**25**	0.787	7	0.080	10
Axon guidance	2.38e-07	38	0.833	12	0.005	23
Calcium signaling pathway	2.39e-07	36	0.822	11	0.014	20
Human cytomegalovirus infection	2.45e-07	41	0.787	17	0.002	27
Longevity regulating pathway	2.58e-07	32	0.787	10	0.010	18
Non-small cell lung cancer	2.90e-07	34	0.904	8	0.002	22
Endometrial cancer	**3.00e-07**	**29**	0.787	8	0.061	13
Oxytocin signaling pathway	3.18e-07	34	0.787	13	0.043	17
Human T-cell leukemia virus 1 infection	3.63e-07	41	0.787	18	0.002	27
Cushing syndrome	3.66e-07	36	0.833	11	0.001	24
EGFR tyrosine kinase inhibitor resistance	**3.68e-07**	**30**	0.787	10	0.155	12
Chemokine signaling pathway	3.68e-07	37	0.787	13	0.008	22
Aldosterone-regulated sodium reabsorption	**3.77e-07**	**21**	0.822	4	0.080	8
Thermogenesis	4.00e-07	39	0.781	19	0.001	26
Choline metabolism in cancer	**4.03e-07**	**32**	0.787	12	0.064	15
Cholinergic synapse	4.03e-07	36	0.787	13	0.032	19
Cocaine addiction	4.58e-07	26	0.787	7	0.043	12
Platinum drug resistance	4.90e-07	31	0.787	12	0.004	19
Melanogenesis	4.90e-07	28	0.873	6	0.014	15
Small cell lung cancer	5.10e-07	36	0.794	12	0.005	22
Serotonergic synapse	5.25e-07	35	0.781	16	0.042	18
Neuroactive ligand-receptor interaction	**5.47e-07**	**38**	0.923	10	0.282	16
NF-kappa B signaling pathway	5.47e-07	30	0.787	9	0.005	18
Cytosolic DNA-sensing pathway	5.53e-07	25	0.787	8	0.015	13
Apoptosis	5.65e-07	38	0.787	15	0.044	20
Tight junction	6.51e-07	38	0.781	19	0.028	21
Ovarian steroidogenesis	**7.50e-07**	**21**	0.833	4	0.499	5
Necroptosis	**8.49e-07**	**33**	0.904	8	0.107	15
Thyroid hormone synthesis	8.49e-07	28	0.781	11	0.017	15
Glutamatergic synapse	8.49e-07	34	0.787	12	0.033	18
MicroRNAs in cancer	8.76e-07	37	0.735	19	8.63e-04	26
Adrenergic signaling in cardiomyocytes	9.31e-07	37	0.787	14	0.035	20
Cytokine-cytokine receptor interaction	9.37e-07	39	0.787	15	0.014	23
Alcoholism	**1.03e-06**	**36**	0.887	10	0.530	13
Vascular smooth muscle contraction	1.05e-06	34	0.849	10	0.035	18
Vasopressin-regulated water reabsorption	1.16e-06	25	0.822	6	0.011	14
Amyotrophic lateral sclerosis	1.25e-06	49	0.477	31	0.048	29
Relaxin signaling pathway	**1.33e-06**	**34**	0.787	14	0.102	16
Ras signaling pathway	**1.41e-06**	**36**	0.822	12	0.125	17
Aldosterone synthesis and secretion	**1.52e-06**	**29**	0.870	7	0.093	13
Bacterial invasion of epithelial cells	**1.52e-06**	**30**	0.796	9	0.217	12
Endocrine and other factor-regulated calcium reabsorption	1.59e-06	24	0.907	4	0.029	12
AGE-RAGE signaling pathway in diabetic complications	**1.71e-06**	**28**	0.787	9	0.070	13
Dopaminergic synapse	**1.84e-06**	**34**	0.787	14	0.112	16
Pertussis	**1.86e-06**	**26**	0.787	8	0.108	11
Primary immunodeficiency	1.90e-06	18	0.787	4	0.008	10
MAPK signaling pathway	1.97e-06	38	0.840	13	0.013	23
Retrograde endocannabinoid signaling	2.04e-06	37	0.787	16	0.014	22
Long-term depression	2.06e-06	26	0.851	6	0.037	13
Prolactin signaling pathway	2.24e-06	28	0.787	10	0.025	15
Regulation of actin cytoskeleton	2.34e-06	32	0.787	11	0.021	18
IL-17 signaling pathway	2.50e-06	29	0.787	10	0.038	15
Cell adhesion molecule (CAM) interaction	**2.53e-06**	**30**	0.787	10	0.244	12
Phospholipase D signaling pathway	2.62e-06	33	0.787	12	0.032	18
Parkinson disease	**2.65e-06**	**40**	0.787	18	0.054	22
Acute myeloid leukemia	2.78e-06	29	0.622	15	0.022	16
Wnt signaling pathway	2.80e-06	31	0.790	10	0.004	20
Shigellosis	**3.17e-06**	**36**	0.781	18	0.060	19
Olfactory transduction	3.33e-06	40	0.822	15	0.035	23
Viral myocarditis	3.33e-06	26	0.787	10	0.006	16
Focal adhesion	3.33e-06	35	0.822	12	0.043	19
Leukocyte transendothelial migration	**3.35e-06**	**28**	0.787	11	0.084	13
Human papillomavirus infection	3.56e-06	42	0.787	19	0.002	29
Complement and coagulation cascades	**3.64e-06**	**25**	0.787	8	0.168	10
Hippo signaling pathway	3.80e-06	38	0.787	17	0.029	22
Renin secretion	4.00e-06	26	0.781	11	0.025	14
Legionellosis	**4.04e-06**	**22**	0.787	7	0.205	8
Protein digestion and absorption	4.08e-06	30	0.781	13	0.003	20
Pathogenic Escherichia coli infection	4.27e-06	38	0.787	18	0.002	26
Pathways of neurodegeneration - multiple diseases	4.92e-06	49	0.622	30	0.024	31
Non-alcoholic fatty liver disease	5.03e-06	38	0.781	20	0.031	22
Collecting duct acid secretion	5.46e-06	18	0.859	3	0.013	10
Alzheimer disease	5.46e-06	42	0.710	24	0.002	29
Toll-like receptor signaling pathway	**6.34e-06**	**28**	0.781	12	0.061	14
VEGF signaling pathway	**6.34e-06**	**26**	0.787	10	0.052	13
cGMP-PKG signaling pathway	6.34e-06	33	0.787	13	0.025	19
Huntington disease	6.34e-06	40	0.781	20	0.007	26
Prion disease	6.74e-06	38	0.781	20	0.035	22
Type I diabetes mellitus	**6.74e-06**	**21**	0.870	4	0.171	8
Phagocytosis	6.79e-06	36	0.822	13	0.014	22
Morphine addiction	**7.98e-06**	**28**	0.879	7	0.064	14
Amoebiasis	9.28e-06	25	0.781	11	0.022	14
PI3K-Akt signaling pathway	9.73e-06	40	0.787	17	0.015	25
Circadian entrainment	**1.04e-05**	**30**	0.787	13	0.080	15
Graft-versus-host disease	**1.04e-05**	**20**	0.851	4	0.136	8
Chagas disease	1.09e-05	29	0.787	12	0.035	16
Salmonella infection	**1.23e-05**	**38**	0.787	17	0.068	21
Hypertrophic cardiomyopathy	1.45e-05	28	0.847	8	0.014	17
Allograft rejection	1.46e-05	20	0.860	4	0.043	10
Insulin secretion	1.57e-05	25	0.787	9	0.048	13
Carbohydrate digestion and absorption	**1.67e-05**	**23**	0.787	8	0.073	11
Yersinia infection	1.93e-05	30	0.787	12	0.019	18
Growth hormone synthesis, secretion and action	**2.03e-05**	**30**	0.787	13	0.098	15
Antigen processing and presentation	**2.17e-05**	**24**	0.851	6	0.064	12
Sphingolipid signaling pathway	2.40e-05	33	0.781	17	0.006	22
cAMP signaling pathway	3.11e-05	33	0.787	13	0.043	19
Dilated cardiomyopathy	3.79e-05	27	0.885	7	0.043	15
GABAergic synapse	**4.56e-05**	**27**	0.847	8	0.076	14
Cardiac muscle contraction	**4.78e-05**	**26**	0.787	9	0.093	13
Gap junction	**5.02e-05**	**29**	0.787	11	0.056	16
Herpes simplex virus 1 infection	7.06e-05	43	0.781	25	0.039	27
Platelet activation	**9.85e-05**	**28**	0.794	10	0.080	15
Rheumatoid arthritis	**9.91e-05**	**21**	0.787	8	0.062	11
Systemic lupus erythematosus	**1.00e-04**	**23**	0.822	7	0.490	8
Autoimmune thyroid disease	**1.25e-04**	**19**	0.822	5	0.057	10
Arrhythmogenic right ventricular cardiomyopathy	**1.35e-04**	**26**	0.885	7	0.076	14
Nicotine addiction	**5.13e-04**	**12**	0.787	3	0.164	5
Regulation of lipolysis in adipocytes	**8.64e-04**	**24**	0.787	10	0.204	12
Maturity onset diabetes of the young	**8.86e-04**	**12**	0.787	4	0.194	5
Intestinal immune network for IgA production	**9.90e-04**	**14**	0.781	7	0.108	7
Staphylococcus aureus infection	**0.003**	**16**	0.787	7	0.490	6

Table S7. NcPath/KEGG pathway enrichment for lncRNA target genes in Cerebellum (Case study 2). Columns and CQRM-exclusive (bold green) marking as in Table S6. Pathways enriched among CQRM-detected lncRNAs are mainly involved in neural circuit formation and cerebellar maturation (axon guidance, Rap1 signaling pathway, circadian rhythm).

Pathway	CQRM adj.p	CQRM n	edgeR adj.p	edgeR n	Wilcoxon adj.p	Wilcoxon n
Taste transduction	5.90e-17	53	0.971	13	5.35e-04	26
Tuberculosis	9.75e-15	59	0.971	16	0.001	31
Inflammatory bowel disease	1.19e-14	49	0.994	7	5.77e-04	25
Ferroptosis	1.19e-14	54	0.663	20	0.001	27
Axon guidance	1.66e-14	60	0.971	19	0.012	28
Circadian rhythm	2.97e-13	40	0.975	6	0.042	14
Vibrio cholerae infection	7.03e-13	42	0.975	7	5.77e-04	22
RIG-I-like receptor signaling pathway	1.67e-12	51	0.975	13	8.43e-05	32
Glucagon signaling pathway	1.88e-12	51	0.971	14	0.017	23
Fat digestion and absorption	2.48e-12	37	0.994	3	0.001	18
Chemical carcinogenesis - DNA adducts	2.48e-12	39	0.994	3	0.012	16
Central carbon metabolism in cancer	3.25e-12	48	0.971	12	0.039	20
NOD-like receptor signaling pathway	3.25e-12	58	0.975	17	4.58e-04	35
Rap1 signaling pathway	4.56e-12	56	0.975	15	0.005	29
Acute myeloid leukemia	5.19e-12	47	0.991	9	0.005	23
Thermogenesis	8.00e-12	58	0.663	24	3.91e-04	36
Transcriptional misregulation in cancer	8.00e-12	62	0.175	30	0.003	35
Melanoma	8.05e-12	50	0.994	9	5.77e-04	29
AMPK signaling pathway	1.30e-11	57	0.971	18	0.022	28
Lipid and atherosclerosis	1.30e-11	55	0.971	18	0.003	30
Gastric acid secretion	1.35e-11	44	0.975	9	0.012	20
Diabetic cardiomyopathy	1.35e-11	58	0.971	19	0.003	32
Bile secretion	1.39e-11	42	0.971	10	0.005	20
Peroxisome	1.39e-11	45	0.971	12	0.003	23
Gastric cancer	1.50e-11	53	0.994	10	6.84e-04	31
Bladder cancer	1.50e-11	42	0.994	7	3.91e-04	25
HIF-1 signaling pathway	1.89e-11	49	0.975	13	0.031	22
B cell receptor signaling pathway	2.01e-11	46	0.994	8	0.009	22
TNF signaling pathway	2.21e-11	54	0.975	14	0.004	29
Non-small cell lung cancer	2.21e-11	50	0.994	9	5.35e-04	30
Chemical carcinogenesis - receptor activation	3.04e-11	57	0.975	17	0.003	32
Phagocytosis	3.37e-11	56	0.971	19	0.006	30
p53 signaling pathway	3.83e-11	51	0.971	16	6.84e-04	30
Hepatitis B	4.98e-11	55	0.975	16	6.95e-04	33
Circadian entrainment	**5.44e-11**	**48**	0.971	15	0.051	21
Phosphatidylinositol signaling system	5.44e-11	45	0.975	11	0.005	23
Thyroid hormone signaling pathway	5.44e-11	50	0.971	16	0.003	27
mTOR signaling pathway	5.44e-11	51	0.975	13	0.003	28
Viral protein interaction with cytokine and cytokine receptor	5.44e-11	32	0.975	5	0.002	16
Epstein-Barr virus infection	5.64e-11	56	0.975	16	0.003	32
Pancreatic cancer	6.07e-11	49	0.994	9	6.84e-04	29
Breast cancer	6.07e-11	52	0.975	13	0.001	30
Hepatocellular carcinoma	6.76e-11	54	0.975	16	5.77e-04	33
Chronic myeloid leukemia	7.11e-11	50	0.994	9	6.12e-04	30
Prostate cancer	7.54e-11	50	0.971	15	0.001	29
Viral carcinogenesis	9.70e-11	59	0.971	21	0.006	33
Small cell lung cancer	1.26e-10	52	0.990	13	3.91e-04	33
Human papillomavirus infection	1.30e-10	62	0.971	24	0.028	33
Pancreatic secretion	1.46e-10	47	0.975	12	0.003	26
MAPK signaling pathway	**1.55e-10**	**56**	0.663	23	0.189	24
JAK-STAT signaling pathway	2.03e-10	49	0.994	9	0.001	29
C-type lectin receptor signaling pathway	2.25e-10	47	0.975	11	0.003	26
Toxoplasmosis	2.39e-10	48	0.994	8	0.004	26
Hedgehog signaling pathway	2.45e-10	35	0.971	8	0.011	16
PI3K-Akt signaling pathway	2.71e-10	60	0.971	22	0.042	31
Glioma	2.71e-10	48	0.994	9	6.84e-04	29
Cushing syndrome	2.71e-10	51	0.975	15	0.005	28
Serotonergic synapse	**2.92e-10**	**50**	0.971	15	0.087	22
Cell cycle	2.92e-10	48	0.971	14	0.001	28
MicroRNAs in cancer	4.34e-10	53	0.663	22	0.001	32
Tight junction	**4.45e-10**	**54**	0.971	18	0.088	25
Huntington disease	5.76e-10	59	0.834	24	0.011	33
Kaposi sarcoma-associated herpesvirus infection	5.82e-10	55	0.975	16	0.002	33
Endocrine resistance	6.18e-10	49	0.990	12	0.001	29
Protein digestion and absorption	**6.18e-10**	**45**	0.990	10	0.056	20
Non-alcoholic fatty liver disease	6.54e-10	56	0.975	18	0.009	31
Thyroid cancer	8.24e-10	35	0.994	5	0.008	17
Neutrophil extracellular trap formation	8.92e-10	50	0.994	11	0.003	29
Alzheimer disease	1.06e-09	61	0.663	27	0.003	37
Insulin resistance	1.31e-09	48	0.975	13	0.007	26
Pathways in cancer	1.43e-09	61	0.663	27	0.006	36
Cellular senescence	1.43e-09	51	0.975	15	0.003	30
Retrograde endocannabinoid signaling	1.48e-09	53	0.971	19	0.035	27
Hematopoietic cell lineage	1.48e-09	41	0.994	8	0.005	22
Human cytomegalovirus infection	1.65e-09	56	0.971	21	0.002	34
ECM-receptor interaction	2.00e-09	45	0.994	7	0.047	21
Fc gamma R-mediated phagosome formation	2.03e-09	44	0.975	11	0.005	24
Cocaine addiction	**2.27e-09**	**36**	0.975	8	0.056	15
Amphetamine addiction	**2.36e-09**	**37**	0.663	13	0.077	15
Influenza A	2.87e-09	51	0.971	18	3.91e-04	34
Endocytosis	2.90e-09	52	0.990	14	0.009	29
Human immunodeficiency virus 1 infection	2.90e-09	53	0.975	17	0.008	30
Olfactory transduction	3.12e-09	57	0.971	22	0.008	33
Thyroid hormone synthesis	**3.79e-09**	**39**	0.975	10	0.150	15
Mitophagy - animal	3.79e-09	36	0.985	7	0.010	18
Adipocytokine signaling pathway	3.97e-09	49	0.994	12	0.009	27
Cholinergic synapse	**4.29e-09**	**49**	0.994	12	0.117	22
Lysosome biogenesis	4.48e-09	38	0.994	5	0.004	21
Longevity regulating pathway	4.48e-09	43	0.994	9	0.005	24
Glutamatergic synapse	**4.77e-09**	**47**	0.971	16	0.068	22
Long-term potentiation	**5.58e-09**	**36**	0.975	9	0.185	13
Chemokine signaling pathway	5.71e-09	50	0.975	14	0.048	25
Autophagy - animal	5.71e-09	44	0.663	17	0.004	25
Platinum drug resistance	6.98e-09	42	0.975	11	5.77e-04	27
Hepatitis C	7.02e-09	52	0.975	16	0.001	33
Basal cell carcinoma	**8.26e-09**	**33**	0.975	7	0.051	14
Fluid shear stress and atherosclerosis	8.95e-09	43	0.990	10	0.003	25
Human T-cell leukemia virus 1 infection	9.90e-09	55	0.971	20	0.009	32
Amyotrophic lateral sclerosis	1.25e-08	67	0.663	34	0.014	41
Epithelial cell signaling in Helicobacter pylori infection	1.39e-08	34	0.975	7	0.026	16
Coronavirus disease	1.70e-08	47	0.994	9	0.011	26
Hippo signaling pathway	**2.15e-08**	**53**	0.971	19	0.105	26
PD-L1 expression and PD-1 checkpoint pathway in cancer	2.35e-08	39	0.971	11	0.033	19
Growth hormone synthesis, secretion and action	**2.49e-08**	**44**	0.994	10	0.223	18
Calcium signaling pathway	**2.49e-08**	**47**	0.893	18	0.065	23
Pathogenic Escherichia coli infection	2.53e-08	53	0.663	23	0.005	32
Notch signaling pathway	2.60e-08	36	0.975	9	0.005	20
Colorectal cancer	**2.70e-08**	**40**	0.994	8	0.078	18
Regulation of actin cytoskeleton	**2.86e-08**	**44**	0.663	19	0.103	20
GnRH signaling pathway	**2.99e-08**	**39**	0.971	12	0.094	17
Estrogen signaling pathway	**3.17e-08**	**45**	0.971	15	0.297	18
Spinocerebellar ataxia	**3.29e-08**	**45**	0.975	14	0.092	21
Fc epsilon RI signaling pathway	3.33e-08	40	0.994	8	0.005	23
Type II diabetes mellitus	**4.42e-08**	**36**	0.975	9	0.069	16
Prion disease	4.80e-08	53	0.872	22	0.020	30
Endometrial cancer	**6.24e-08**	**37**	0.994	6	0.063	17
Proteoglycans in cancer	6.61e-08	46	0.663	19	0.039	24
Herpes simplex virus 1 infection	**6.89e-08**	**62**	0.663	30	0.064	35
PPAR signaling pathway	6.99e-08	44	0.994	11	0.002	28
Th1 and Th2 cell differentiation	**7.02e-08**	**38**	0.971	11	0.055	18
Adrenergic signaling in cardiomyocytes	7.02e-08	49	0.834	20	0.042	26
Primary immunodeficiency	7.03e-08	24	0.994	2	0.045	10
Melanogenesis	7.63e-08	36	0.975	9	0.014	19
Inflammatory mediator regulation of TRP channels	7.73e-08	36	0.971	11	0.028	18
Wnt signaling pathway	**7.76e-08**	**42**	0.663	17	0.115	19
Measles	7.91e-08	46	0.971	17	0.001	30
Insulin signaling pathway	8.59e-08	46	0.994	11	0.042	24
IL-17 signaling pathway	**9.02e-08**	**39**	0.990	9	0.078	18
Parkinson disease	9.69e-08	54	0.663	24	0.040	30
Cholesterol metabolism	1.36e-07	34	0.994	6	0.002	21
Parathyroid hormone synthesis, secretion and action	**1.57e-07**	**39**	0.975	10	0.089	18
Oxytocin signaling pathway	**1.57e-07**	**43**	0.834	17	0.117	20
Renin secretion	**1.63e-07**	**35**	0.971	10	0.389	12
Cytokine-cytokine receptor interaction	1.67e-07	51	0.975	16	0.004	32
Vasopressin-regulated water reabsorption	**2.30e-07**	**32**	0.975	8	0.051	15
Vascular smooth muscle contraction	**2.35e-07**	**44**	0.663	19	0.074	22
Shigellosis	2.35e-07	48	0.663	21	0.004	30
Ras signaling pathway	**2.47e-07**	**47**	0.971	17	0.077	24
Cytosolic DNA-sensing pathway	**3.09e-07**	**31**	0.975	7	0.067	14
VEGF signaling pathway	**3.11e-07**	**35**	0.975	9	0.051	17
Relaxin signaling pathway	**3.12e-07**	**44**	0.971	17	0.184	20
Neurotrophin signaling pathway	3.37e-07	40	0.994	8	0.003	25
Pathways of neurodegeneration - multiple diseases	**3.67e-07**	**66**	0.663	35	0.051	40
EGFR tyrosine kinase inhibitor resistance	3.82e-07	37	0.995	5	0.039	19
Natural killer cell mediated cytotoxicity	3.97e-07	40	0.975	12	0.006	24
Renal cell carcinoma	**4.19e-07**	**36**	0.975	9	0.120	16
Aldosterone synthesis and secretion	**4.82e-07**	**37**	0.663	15	0.105	17
cGMP-PKG signaling pathway	**5.50e-07**	**44**	0.971	17	0.208	20
Apoptosis	5.68e-07	48	0.971	19	0.011	29
NF-kappa B signaling pathway	6.36e-07	37	0.975	10	0.026	20
Cortisol synthesis and secretion	**6.44e-07**	**33**	0.971	10	0.089	15
Insulin secretion	**6.68e-07**	**34**	0.663	14	0.077	16
Long-term depression	**6.98e-07**	**33**	0.975	8	0.056	16
Salivary secretion	8.87e-07	39	0.975	12	0.005	24
Leukocyte transendothelial migration	**9.24e-07**	**36**	0.975	10	0.092	17
Choline metabolism in cancer	9.24e-07	39	0.994	8	0.019	22
Signaling pathways regulating pluripotency of stem cells	9.75e-07	40	0.971	14	0.009	24
Dopaminergic synapse	**1.43e-06**	**43**	0.971	16	0.286	19
Synaptic vesicle cycle	1.47e-06	31	0.971	10	0.019	17
FoxO signaling pathway	**1.56e-06**	**41**	0.991	11	0.077	21
Prolactin signaling pathway	1.67e-06	35	0.975	9	0.029	19
Apelin signaling pathway	**1.83e-06**	**43**	0.872	18	0.211	20
T cell receptor signaling pathway	**2.03e-06**	**38**	0.971	14	0.083	19
Antigen processing and presentation	**2.16e-06**	**32**	0.999	2	0.148	14
Amoebiasis	**3.36e-06**	**32**	0.971	10	0.064	16
AGE-RAGE signaling pathway in diabetic complications	**3.64e-06**	**34**	0.994	7	0.075	17
GnRH secretion	**4.16e-06**	**36**	0.975	11	0.209	16
Leishmaniasis	4.19e-06	31	0.994	5	0.014	18
cAMP signaling pathway	4.32e-06	44	0.971	17	0.044	25
Phospholipase D signaling pathway	4.43e-06	41	0.975	13	0.007	26
Alcoholism	**4.43e-06**	**44**	0.975	14	0.425	19
Arrhythmogenic right ventricular cardiomyopathy	**5.98e-06**	**36**	0.834	15	0.781	11
ErbB signaling pathway	**7.99e-06**	**35**	0.975	10	0.078	18
Platelet activation	8.31e-06	38	0.975	12	0.030	22
Adherens junction	**8.65e-06**	**30**	0.990	7	0.117	14
Sphingolipid signaling pathway	8.76e-06	43	0.975	15	0.007	28
Salmonella infection	8.99e-06	49	0.663	25	0.008	32
Necroptosis	9.17e-06	39	0.971	15	0.008	25
TGF-beta signaling pathway	**9.33e-06**	**32**	0.971	11	0.084	16
Legionellosis	1.20e-05	26	0.994	5	0.018	15
Focal adhesion	**1.22e-05**	**43**	0.971	16	0.323	20
Gap junction	**1.39e-05**	**38**	0.971	14	0.059	21
Collecting duct acid secretion	**1.44e-05**	**21**	0.994	2	0.064	10
GABAergic synapse	**1.65e-05**	**35**	0.971	12	0.151	17
Toll-like receptor signaling pathway	1.73e-05	34	0.994	8	0.026	20
Th17 cell differentiation	**2.17e-05**	**33**	0.975	10	0.057	18
Malaria	**2.62e-05**	**37**	0.994	7	0.052	21
Cardiac muscle contraction	**3.42e-05**	**33**	0.971	12	0.104	17
Rheumatoid arthritis	**4.07e-05**	**27**	0.995	3	0.196	12
Yersinia infection	4.51e-05	37	0.994	9	0.022	23
Pertussis	4.85e-05	29	0.994	6	0.016	18
Phototransduction	**5.59e-05**	**17**	0.979	3	0.267	6
Morphine addiction	**6.11e-05**	**33**	0.971	12	0.124	17
Endocrine and other factor-regulated calcium reabsorption	**6.56e-05**	**26**	0.971	9	0.170	12
Osteoclast differentiation	**9.09e-05**	**37**	0.975	12	0.051	22
Regulation of lipolysis in adipocytes	**9.17e-05**	**33**	0.975	10	0.213	16
Dilated cardiomyopathy	**9.39e-05**	**33**	0.971	14	0.425	14
Antifolate resistance	**9.54e-05**	**25**	0.975	7	0.051	14
Ovarian steroidogenesis	1.25e-04	21	0.748	9	0.019	13
Renin-angiotensin system	1.52e-04	17	0.990	3	0.028	10
Complement and coagulation cascades	**2.02e-04**	**27**	0.994	5	0.673	9
Cell adhesion molecule (CAM) interaction	**2.03e-04**	**33**	0.985	10	0.601	13
Neuroactive ligand-receptor interaction	**2.04e-04**	**42**	0.920	20	0.360	21
Proximal tubule bicarbonate reclamation	2.22e-04	18	0.975	5	0.011	12
Viral myocarditis	2.32e-04	28	0.975	8	0.006	20
Carbohydrate digestion and absorption	5.46e-04	25	0.971	10	0.031	16
Chagas disease	**6.61e-04**	**32**	0.990	10	0.129	18
Bacterial invasion of epithelial cells	**7.02e-04**	**31**	0.994	8	0.330	15
Nicotine addiction	**9.99e-04**	**14**	0.994	2	--	--
Mineral absorption	**0.001**	**24**	0.971	9	0.220	12
Hypertrophic cardiomyopathy	**0.001**	**30**	0.971	13	0.555	13
Systemic lupus erythematosus	**0.003**	**25**	0.999	3	0.489	11
Aldosterone-regulated sodium reabsorption	**0.004**	**17**	0.971	6	0.430	7
Type I diabetes mellitus	**0.012**	**18**	0.991	5	0.350	9
Graft-versus-host disease	**0.015**	**17**	0.994	3	0.425	8
Vitamin digestion and absorption	**0.017**	**14**	0.975	4	0.196	8
Allograft rejection	**0.018**	**17**	0.994	3	0.450	8

Table S8. NcPath/KEGG pathway enrichment for lncRNA target genes in Testis (Case study 2). Columns and CQRM-exclusive (bold green) marking as in Table S6. Pathways enriched among CQRM-detected lncRNAs are mainly involved in endocrine and reproductive signaling (insulin secretion, thyroid hormone signaling pathway, oxytocin signaling pathway). Across all three organs, the enriched pathways are relevant to the respective organ's developmental biology, and the distinct patterns per organ highlight tissue-specific regulatory roles of the detected lncRNAs.

Pathway	CQRM adj.p	CQRM n	edgeR adj.p	edgeR n	Wilcoxon adj.p	Wilcoxon n
Transcriptional misregulation in cancer	1.28e-06	119	0.006	70	6.41e-11	107
Pancreatic secretion	1.06e-04	80	0.997	29	1.07e-11	82
Insulin secretion	2.48e-04	64	0.997	21	4.03e-09	63
Ferroptosis	2.83e-04	78	1.000	24	1.21e-13	87
Thermogenesis	2.83e-04	99	1.000	36	1.53e-10	97
Serotonergic synapse	4.29e-04	85	0.997	34	1.93e-10	86
Calcium signaling pathway	4.29e-04	86	0.997	40	1.93e-10	87
NOD-like receptor signaling pathway	7.18e-04	95	1.000	34	4.76e-11	97
Amyotrophic lateral sclerosis	7.45e-04	138	0.997	79	1.66e-07	122
Peroxisome	7.93e-04	66	1.000	14	3.85e-08	65
Vascular smooth muscle contraction	0.001	81	0.997	35	6.72e-11	86
Gastric acid secretion	0.001	63	0.997	25	1.77e-17	84
Cholinergic synapse	0.001	85	0.997	39	1.69e-11	91
Taste transduction	0.001	65	1.000	18	9.95e-14	79
Thyroid hormone signaling pathway	0.001	79	0.997	29	1.40e-09	81
Shigellosis	0.001	90	0.997	41	7.62e-08	86
Oxytocin signaling pathway	0.001	77	0.997	32	1.39e-11	85
Vibrio cholerae infection	0.001	53	1.000	11	1.21e-13	68
Human papillomavirus infection	0.001	112	0.997	48	1.94e-11	113
Nicotine addiction	0.001	29	0.997	9	0.009	20
Protein digestion and absorption	0.001	71	1.000	23	1.77e-17	92
Pathways of neurodegeneration - multiple diseases	0.001	140	0.997	80	5.51e-06	121
Salivary secretion	0.001	71	0.997	37	1.56e-08	72
cGMP-PKG signaling pathway	0.001	82	0.997	37	1.86e-11	89
Central carbon metabolism in cancer	0.001	68	0.997	29	1.24e-10	75
Parathyroid hormone synthesis, secretion and action	0.001	67	0.997	29	1.72e-10	74
MicroRNAs in cancer	0.001	89	1.000	26	1.21e-13	101
AMPK signaling pathway	0.001	92	0.997	38	2.63e-10	95
Bile secretion	0.001	57	0.997	24	5.80e-12	69
Retrograde endocannabinoid signaling	0.001	91	0.997	36	9.91e-10	93
Th17 cell differentiation	0.002	63	0.997	29	2.22e-06	60
Phospholipase D signaling pathway	0.002	78	0.997	32	2.19e-10	84
Pathways in cancer	0.002	112	0.997	51	5.80e-12	116
Axon guidance	0.002	88	0.997	38	5.53e-13	99
Focal adhesion	0.002	85	0.997	36	2.63e-10	90
Circadian entrainment	0.002	72	0.997	33	7.32e-12	83
Cytokine-cytokine receptor interaction	0.002	95	0.997	43	1.33e-06	88
Platelet activation	0.002	72	0.997	29	1.21e-13	87
cAMP signaling pathway	0.002	85	0.997	37	1.46e-09	88
Phosphatidylinositol signaling system	0.002	65	0.997	25	1.39e-11	76
Adrenergic signaling in cardiomyocytes	0.002	88	0.997	37	6.47e-14	102
Sphingolipid signaling pathway	0.002	84	0.997	38	1.07e-11	93
Acute myeloid leukemia	0.002	65	0.997	27	2.81e-06	62
GABAergic synapse	0.002	66	0.997	30	1.23e-10	75
Chemical carcinogenesis - receptor activation	0.002	92	0.997	40	1.16e-09	95
Glutamatergic synapse	0.002	77	0.997	33	4.76e-11	86
Non-small cell lung cancer	0.002	74	1.000	27	1.28e-08	77
mTOR signaling pathway	0.002	78	0.997	30	6.64e-10	84
Notch signaling pathway	0.002	55	0.997	20	1.07e-11	68
Human cytomegalovirus infection	0.002	97	0.997	44	1.86e-11	104
Rap1 signaling pathway	0.003	85	1.000	32	1.27e-10	92
Cortisol synthesis and secretion	0.003	54	0.997	19	3.83e-09	61
Proteoglycans in cancer	0.003	79	0.997	35	5.89e-07	77
Long-term potentiation	0.003	52	0.997	23	8.35e-10	61
Alzheimer disease	0.003	109	1.000	46	1.37e-07	104
Cushing syndrome	0.003	80	1.000	30	2.54e-13	95
Morphine addiction	0.003	63	0.997	32	5.80e-12	77
Renin secretion	0.004	55	1.000	18	6.72e-11	67
Amphetamine addiction	0.004	52	0.997	20	1.31e-07	56
Gastric cancer	0.004	79	0.997	32	3.75e-09	84
Amoebiasis	0.004	54	0.997	23	1.31e-11	68
Influenza A	0.004	84	0.997	41	2.45e-08	86
Inflammatory bowel disease	0.004	58	0.997	28	1.94e-10	69
Bladder cancer	0.004	54	1.000	12	2.45e-08	60
Aldosterone synthesis and secretion	0.004	61	0.997	25	2.50e-09	69
Endocrine resistance	0.004	76	0.997	33	1.24e-09	83
Hepatocellular carcinoma	0.004	84	0.997	38	1.93e-10	92
Alcoholism	0.004	82	0.997	42	2.20e-07	82
GnRH signaling pathway	0.005	60	0.997	22	7.47e-09	67
Huntington disease	0.005	101	0.997	44	4.21e-06	94
Cocaine addiction	0.005	49	0.997	23	1.45e-09	59
PI3K-Akt signaling pathway	0.005	102	0.997	52	2.78e-12	112
Parkinson disease	0.005	98	1.000	39	3.75e-07	95
Spinocerebellar ataxia	0.006	73	0.997	35	5.22e-07	74
Pathogenic Escherichia coli infection	0.006	92	0.997	40	9.42e-09	95
Neuroactive ligand-receptor interaction	0.006	86	0.997	46	1.18e-07	87
Dopaminergic synapse	0.006	76	0.997	41	1.24e-09	84
Thyroid cancer	0.006	45	1.000	13	1.13e-07	51
Small cell lung cancer	0.006	79	0.997	34	1.58e-10	89
Cellular senescence	0.007	81	0.997	35	4.16e-09	87
Coronavirus disease	0.007	76	0.997	38	2.22e-07	78
Circadian rhythm	0.007	43	1.000	11	3.57e-09	53
Prion disease	0.007	93	1.000	31	5.59e-06	88
Inflammatory mediator regulation of TRP channels	0.007	54	0.997	21	9.55e-12	70
Chemokine signaling pathway	0.007	81	0.997	38	1.63e-10	91
Basal cell carcinoma	0.007	44	0.997	17	1.81e-07	50
ECM-receptor interaction	0.007	67	0.997	30	6.55e-11	80
Viral carcinogenesis	0.007	96	0.997	46	3.83e-09	100
Apelin signaling pathway	0.007	76	0.997	38	4.75e-12	91
Hepatitis B	0.007	84	0.997	35	9.55e-12	97
Breast cancer	0.008	77	1.000	31	2.45e-08	82
Regulation of actin cytoskeleton	0.010	69	0.997	37	3.75e-08	75
Non-alcoholic fatty liver disease	0.010	91	1.000	35	1.35e-05	86
Phagocytosis	0.010	85	0.997	42	1.44e-05	81
Glucagon signaling pathway	0.011	67	0.997	33	1.27e-10	80
Herpes simplex virus 1 infection	0.011	117	0.997	59	3.62e-07	112
Renin-angiotensin system	0.011	27	0.997	8	5.61e-06	31
Estrogen signaling pathway	0.012	71	0.997	41	8.36e-09	79
Fluid shear stress and atherosclerosis	0.012	64	0.997	32	2.20e-07	69
B cell receptor signaling pathway	0.012	60	0.997	27	3.60e-06	62
Measles	0.013	75	0.997	40	3.35e-08	81
Tuberculosis	0.013	77	0.997	35	7.07e-11	90
Adipocytokine signaling pathway	0.013	76	0.997	37	3.22e-05	73
Platinum drug resistance	0.013	61	0.997	24	2.35e-09	72
Signaling pathways regulating pluripotency of stem cells	0.013	66	0.997	34	1.84e-08	74
Synaptic vesicle cycle	0.013	47	1.000	13	5.19e-11	63
IL-17 signaling pathway	0.013	59	0.997	25	6.63e-09	69
p53 signaling pathway	0.014	72	1.000	18	2.10e-09	82
Gap junction	0.014	67	0.997	30	4.36e-08	74
Dilated cardiomyopathy	0.014	60	0.997	29	1.59e-08	69
Cytosolic DNA-sensing pathway	0.015	44	0.997	22	2.91e-07	51
Hedgehog signaling pathway	0.015	41	0.997	16	5.59e-10	55
Hepatitis C	0.015	84	0.997	38	1.84e-08	90
Pancreatic cancer	0.015	68	0.997	28	4.98e-08	75
Epithelial cell signaling in Helicobacter pylori infection	0.015	45	0.997	17	1.24e-09	58
Th1 and Th2 cell differentiation	0.015	56	0.997	33	1.43e-07	63
Proximal tubule bicarbonate reclamation	0.015	29	0.997	10	0.002	27
Vitamin digestion and absorption	0.015	30	0.997	12	8.83e-09	41
Longevity regulating pathway	0.015	62	0.997	26	3.08e-09	73
Thyroid hormone synthesis	0.015	53	0.997	26	6.55e-11	69
Choline metabolism in cancer	0.015	63	0.997	28	3.85e-08	71
PPAR signaling pathway	0.016	69	0.997	32	2.45e-08	77
Human T-cell leukemia virus 1 infection	0.016	92	0.997	43	1.74e-08	97
Lysosome biogenesis	0.016	51	1.000	15	5.59e-10	65
Endocrine and other factor-regulated calcium reabsorption	0.016	43	0.997	19	1.09e-10	59
GnRH secretion	0.016	59	0.997	33	3.97e-06	62
Prolactin signaling pathway	0.016	55	0.997	23	3.90e-08	64
RIG-I-like receptor signaling pathway	0.017	65	0.997	34	4.61e-11	81
Long-term depression	0.017	49	1.000	17	4.26e-09	61
Tight junction	0.017	83	0.997	37	7.06e-06	82
Regulation of lipolysis in adipocytes	0.017	59	0.997	28	6.33e-09	70
MAPK signaling pathway	0.017	86	0.997	40	9.79e-09	93
Hippo signaling pathway	0.017	88	0.997	41	3.31e-09	96
Prostate cancer	0.017	70	0.997	30	5.65e-09	80
HIF-1 signaling pathway	0.017	65	1.000	26	1.77e-09	77
Adherens junction	0.018	47	0.997	19	2.87e-06	52
Lipid and atherosclerosis	0.018	78	0.997	36	2.80e-09	88
Melanogenesis	0.018	51	0.997	27	1.22e-13	74
Chemical carcinogenesis - DNA adducts	0.018	41	1.000	10	1.85e-08	52
NF-kappa B signaling pathway	0.018	57	0.997	25	2.69e-07	64
Renal cell carcinoma	0.018	54	0.997	24	6.48e-08	63
Aldosterone-regulated sodium reabsorption	0.019	32	0.997	11	1.84e-05	36
Endocytosis	0.020	81	0.997	40	2.34e-09	91
Olfactory transduction	0.020	94	1.000	41	5.83e-07	95
Glioma	0.020	67	1.000	26	4.99e-09	78
Growth hormone synthesis, secretion and action	0.020	66	0.997	30	6.87e-08	74
Pertussis	0.020	48	0.997	19	1.60e-06	54
Relaxin signaling pathway	0.020	71	0.997	37	4.76e-11	87
Neurotrophin signaling pathway	0.020	62	0.997	31	2.58e-06	66
Fat digestion and absorption	0.021	37	1.000	8	1.30e-06	44
Leukocyte transendothelial migration	0.021	55	0.997	24	1.08e-06	61
Chronic myeloid leukemia	0.022	69	0.997	30	9.89e-10	82
C-type lectin receptor signaling pathway	0.022	64	0.997	30	8.79e-09	75
Cholesterol metabolism	0.024	47	0.997	21	8.73e-10	62
Hematopoietic cell lineage	0.024	54	0.997	26	6.64e-10	69
Yersinia infection	0.026	65	0.997	35	6.17e-08	74
Carbohydrate digestion and absorption	0.027	44	0.997	20	3.69e-06	50
Arrhythmogenic right ventricular cardiomyopathy	0.027	58	0.997	28	2.83e-07	66
Bacterial invasion of epithelial cells	0.029	58	0.997	30	3.08e-07	66
Colorectal cancer	0.031	56	0.997	25	8.24e-08	66
Ovarian steroidogenesis	0.032	32	0.997	12	3.91e-09	46
Diabetic cardiomyopathy	0.032	84	1.000	37	1.12e-08	93
Leishmaniasis	0.033	46	0.997	25	2.19e-06	53
AGE-RAGE signaling pathway in diabetic complications	0.034	52	0.997	26	1.59e-06	59
JAK-STAT signaling pathway	0.034	67	0.997	29	4.16e-08	77
Toxoplasmosis	0.034	65	0.997	31	1.43e-12	87
Insulin signaling pathway	0.039	71	0.997	33	1.91e-07	79
Cell cycle	0.040	65	1.000	24	4.40e-10	81
Human immunodeficiency virus 1 infection	0.041	81	0.997	39	1.08e-09	94
T cell receptor signaling pathway	0.041	59	0.997	29	5.65e-05	61
TNF signaling pathway	0.043	74	0.997	34	3.21e-05	75
Ras signaling pathway	0.045	75	0.997	36	7.79e-08	84
Insulin resistance	0.047	67	0.997	35	1.69e-08	79
Wnt signaling pathway	0.047	61	0.997	37	9.91e-10	77
Apoptosis	0.047	79	0.997	38	2.80e-06	83
Mineral absorption	0.047	42	0.997	20	5.42e-09	57
Type II diabetes mellitus	0.050	47	0.997	20	3.40e-08	60
Melanoma	0.051	62	0.997	27	3.40e-08	74
PD-L1 expression and PD-1 checkpoint pathway in cancer	0.051	52	0.997	29	4.12e-06	59
Hypertrophic cardiomyopathy	0.051	56	0.997	26	1.25e-07	67
Kaposi sarcoma-associated herpesvirus infection	0.052	82	0.997	41	4.16e-09	94
Mitophagy - animal	0.052	43	0.997	18	2.65e-07	54
Autophagy - animal	0.057	60	0.997	34	7.84e-10	77
Antifolate resistance	0.057	38	1.000	14	9.13e-09	53
Neutrophil extracellular trap formation	0.061	70	0.997	40	4.90e-09	84
Viral myocarditis	0.063	46	0.997	28	2.83e-10	65
Fc epsilon RI signaling pathway	0.064	54	0.997	23	2.61e-07	65
EGFR tyrosine kinase inhibitor resistance	0.064	52	0.997	25	2.91e-07	63
Chagas disease	0.074	57	0.997	33	2.45e-08	71
Natural killer cell mediated cytotoxicity	0.076	58	1.000	22	5.79e-11	79
TGF-beta signaling pathway	0.076	47	0.997	26	7.82e-09	63
ErbB signaling pathway	0.076	53	0.997	29	1.00e-06	63
Cell adhesion molecule (CAM) interaction	0.076	56	0.997	31	1.80e-06	65
Osteoclast differentiation	0.077	63	0.997	32	2.20e-07	74
Fc gamma R-mediated phagosome formation	0.078	57	0.997	25	1.28e-08	72
Legionellosis	0.083	35	0.997	16	6.90e-05	41
Cardiac muscle contraction	0.096	51	0.997	22	9.08e-07	62
Necroptosis	0.106	61	1.000	27	5.15e-08	75
Endometrial cancer	0.107	47	0.997	22	2.83e-07	60
Phototransduction	0.109	20	0.997	9	3.52e-07	32
Epstein-Barr virus infection	0.114	77	0.997	39	3.14e-08	90
Collecting duct acid secretion	0.114	25	0.997	9	1.30e-05	34
Salmonella infection	0.124	84	0.997	44	1.83e-07	94
Primary immunodeficiency	0.126	23	1.000	5	2.86e-07	36
Malaria	0.130	58	0.997	33	5.33e-05	64
Systemic lupus erythematosus	0.139	43	0.997	29	8.09e-06	53
Toll-like receptor signaling pathway	0.195	49	0.997	24	7.26e-06	60
Vasopressin-regulated water reabsorption	0.203	37	0.997	21	1.93e-09	58
Rheumatoid arthritis	0.211	36	0.997	18	6.42e-06	48
VEGF signaling pathway	0.217	43	0.997	24	1.35e-05	54
Complement and coagulation cascades	0.283	38	0.997	20	0.002	43
FoxO signaling pathway	0.361	56	0.997	34	4.30e-07	73
Type I diabetes mellitus	0.387	28	0.997	19	0.007	33
Maturity onset diabetes of the young	0.414	16	1.000	4	9.08e-04	24
Antigen processing and presentation	0.437	37	0.997	18	2.01e-05	51
Viral protein interaction with cytokine and cytokine receptor	0.454	23	0.997	12	6.55e-09	45
Autoimmune thyroid disease	0.617	26	0.997	19	0.003	35
Graft-versus-host disease	0.756	22	0.997	17	0.043	28
Allograft rejection	0.793	22	0.997	18	0.007	32
Intestinal immune network for IgA production	0.884	15	0.997	14	0.014	25
